# Imiquimod Boosts Interferon Response, and Decreases ACE2 and Pro-Inflammatory Response of Human Bronchial Epithelium in Asthma

**DOI:** 10.3389/fimmu.2021.743890

**Published:** 2021-12-07

**Authors:** Juan José Nieto-Fontarigo, Sofia Tillgren, Samuel Cerps, Asger Sverrild, Morten Hvidtfeldt, Sangeetha Ramu, Mandy Menzel, Adam Frederik Sander, Celeste Porsbjerg, Lena Uller

**Affiliations:** ^1^ Department of Experimental Medical Science, Lund University, Lund, Sweden; ^2^ Department of Respiratory Medicine, University Hospital Bispebjerg, Copenhagen, Denmark; ^3^ Department for Immunology and Microbiology, Faculty of Health and Medical Sciences, Centre for Medical Parasitology, University of Copenhagen, Copenhagen, Denmark; ^4^ Department of Infectious Disease, Copenhagen University Hospital, Copenhagen, Denmark

**Keywords:** asthma, anti-viral drug, SARS – CoV – 2, imiquimod, COVID-19, TLR7 agonist

## Abstract

**Background:**

Both anti-viral and anti-inflammatory bronchial effects are warranted to treat viral infections in asthma. We sought to investigate if imiquimod, a TLR7 agonist, exhibits such dual actions in *ex vivo* cultured human bronchial epithelial cells (HBECs), targets for SARS-CoV-2 infectivity.

**Objective:**

To investigate bronchial epithelial effects of imiquimod of potential importance for anti-viral treatment in asthmatic patients.

**Methods:**

Effects of imiquimod alone were examined in HBECs from healthy (N=4) and asthmatic (N=18) donors. Mimicking SARS-CoV-2 infection, HBECs were stimulated with poly(I:C), a dsRNA analogue, or SARS-CoV-2 spike-protein 1 (SP1; receptor binding) with and without imiquimod treatment. Expression of SARS-CoV-2 receptor (ACE2), pro-inflammatory and anti-viral cytokines were analyzed by RT-qPCR, multiplex ELISA, western blot, and Nanostring and proteomic analyses.

**Results:**

Imiquimod reduced ACE2 expression at baseline and after poly(I:C) stimulation. Imiquimod also reduced poly(I:C)-induced pro-inflammatory cytokines including IL-1β, IL-6, IL-8, and IL-33. Furthermore, imiquimod increased IFN-β expression, an effect potentiated in presence of poly(I:C) or SP1. Multiplex mRNA analysis verified enrichment in type-I IFN signaling concomitant with suppression of cytokine signaling pathways induced by imiquimod in presence of poly(I:C). Exploratory proteomic analyses revealed potentially protective effects of imiquimod on infections.

**Conclusion:**

Imiquimod triggers viral resistance mechanisms in HBECs by decreasing ACE2 and increasing IFN-β expression. Additionally, imiquimod improves viral infection tolerance by reducing viral stimulus-induced epithelial cytokines involved in severe COVID-19 infection. Our imiquimod data highlight feasibility of producing pluripotent drugs potentially suited for anti-viral treatment in asthmatic subjects.

## Introduction

Asthma is a chronic inflammatory disease affecting more than 300 million people worldwide (1). Acute exacerbations of asthma are the major cause of disease worsening and morbidity, and they are responsible for increasing health care burden (2). Rhinovirus infection is the major trigger for asthma exacerbation (2). However, other respiratory viruses such as respiratory syncytial virus (RSV), influenza A or coronaviruses have been also related to an aggravation of the disease (2). For example, intensive care admissions and mortality of patients with H1N1 influenza A was higher in patients with asthma during 2009 H1N1 pandemic (3).

In December 2019, a novel coronavirus disease, named coronavirus disease-19 (COVID-19), emerged in the Hubei province of China (4). This disease is caused by severe acute respiratory syndrome-related coronavirus 2 (SARS-CoV-2) (5). Although the first epidemiologic studies could not find an association of asthma with an increase risk to suffer from COVID-19, novel data from large cohorts evidenced a higher prevalence of asthma in patients with COVID-19 compared to the general population (6). Asthma was also associated to a longer duration of severe COVID-19 disease (7). Furthermore, several studies demonstrated that severe asthma is a risk factor for developing severe COVID-19 (8, 9). The high infection and mortality rates, in combination with the high economic impact of COVID-19, underpinned an unprecedented global search for efficient treatments and successful developments of vaccines. However, there is still a need for new drug opportunities to treat SARS-CoV-2 infection, especially in patients with a high risk to develop a severe sickness such as asthmatic subjects.

Similar to rhinoviruses, SARS-CoV-2 is a single stranded RNA (ssRNA) virus (10). SARS-CoV-2 mainly infects human cells through the high affinity interaction of the receptor binding domain (RBD) of the spike protein 1 (SP1) with the angiotensin converting enzyme II (ACE2) protein on host cells (11, 12). ACE2 is widely expressed in different cell types and tissues including bronchial and upper airway epithelial cells, representing the main port of entry by SARS-CoV-2 (11, 13–15). In addition to ACE2, the host serine protease TMPRSS2, responsible for fusion of the viral and host cell membranes by cleaving S protein at the S1/S2 and S2´sites, is essential for viral entry (11). Hence, investigating the expression of both ACE2 and TMPRSS2 in the bronchial epithelium may contribute to understand the infectivity and actions of SARS-CoV-2 and assist in the search of pharmacological opportunities to combat COVID-19.

Following cell entry, both rhinovirus and SARS-CoV-2 virus replicate and produce intermediate double stranded RNA (dsRNA) molecules (16, 17). dsRNA molecules activate pattern recognition receptors (PRRs) in host cells including Toll-like Receptor (TLR)3, Melanoma Differentiation-Associated protein 5 (MDA5) and Retinoic Acid-Inducible Gene I (RIG-I) (17, 18). Activation of PRRs results in the activation of two important downstream transcriptional factors, NF-κB and Interferon Regulatory Factors (IRFs) leading to production of cytokines or chemokines (such as IL-6, TNF-α, IL-8) and type-I and type-III interferons (IFN; IFN-β and IFN-λ), respectively (17). These molecules represent early responses to viral infection, followed later by anti-viral T-cell responses (17, 19). In COVID-19, a low induction of type-I and type-III IFNs has been described (20, 21). A dysregulation of IFN pathways at early stages of COVID-19 is thought to contribute to uncontrolled viral replication and subsequent dramatic immune responses at later stages (20–23). In addition, asthmatic patients have been described to have an impaired anti-viral IFN response (24, 25), which could predispose these patients to be more susceptible to viral infections. Therefore, one of the desired treatment strategies in order to improve the outcome of viral infection in these patients is the increase of type I IFN responses in the bronchial epithelium.

A second common feature of asthma exacerbations after viral infection and severe COVID-19 is the exaggerated pro-inflammatory response of the bronchial epithelium to virus (17, 26). In this regard, glucocorticoids are an acknowledged treatment of both asthma and severe COVID-19 due to their broad anti-inflammatory effects (27–29). Indeed, inhaled budesonide administered in the early stages of COVID-19 reduced the need for urgent medical care and improved the recovery time (30). However, due to a potential to decrease the anti-viral type I interferon response (31–34), a high dose of glucocorticoids could be harmful if they are given at the peak of viral replication (34). It is, therefore, when the immunopathological features of the illness become more evident that the glucocorticoid effect could be most beneficial, creating a window of opportunity vital to clinical effect (27, 34). The question arises whether drugs can be found that both improve anti-viral responses and reduce severity of pro-inflammatory responses.

Previous attempts to discover pharmacological opportunities to improve both anti-viral defense and reducing cytokine inflammation have been proven to be difficult to combine in one single molecule [(35), and unpublished observations]. Literature data, however, suggest that imiquimod, an imidazo-quinoline used as an immune response modifier, could be an interesting compound to explore further in regards of combined anti-viral and anti-inflammatory actions. Imiquimod is a TLR7 agonist clinically approved as topical treatment of viral and tumoral skin conditions. Imiquimod may activate the production of type-I and III interferons as well as the NF-κB pathway (36), although results vary (37, 38) and effects on human airway epithelium seem unexplored. Imiquimod has been considered as treatment for allergic diseases like asthma due to its inhibition of allergic type 2 inflammation and favoring of type 1 responses (39, 40). Focusing on potential anti-allergic efficacy, local airway treatment with other TLR7 agonist have been employed in clinical trials in asthma and allergy (41, 42). Interestingly, intranasal administration of imiquimod has been demonstrated to protect against Influenza A virus in mice, reducing viral replication, neutrophil infiltration, and lung dysfunction (37). Based on this observation, and observations on potential anti-viral actions in a variety of cell systems, imiquimod has already been suggested as a potential therapy in early phase COVID-19 disease (43–45). However, studies have not focused on major target cells for primary site of infectivity of SARS-CoV-2, the human airway epithelium. Little is thus known about effects of imiquimod on human primary bronchial epithelial cells (HBECs). Considering the occurrence of reduced anti-viral resistance in asthma, demonstrated as reduced interferon production at viral infection (46), and the increasing evidence demonstrating that severe asthma is a risk factor for COVID-19 related admission to intensive care (8, 9), studies of imiquimod in HBECs from asthmatic patients are of particular interest.

Our study explores effects of imiquimod on ACE2 and TMPRSS2 expression in HBECs stimulated with both the TLR3 agonist poly(I:C) (SARS-CoV-2 replication mimic) or the recombinant SP1 from SARS-CoV-2 (RBD). In addition, effects of imiquimod on anti-viral and anti-inflammatory responses were examined by using multiplex ELISA and gene expression analyses, and non-targeted proteomics. We also used siRNA technology to shed light on the potential mechanisms of action of imiquimod in HBECs.

## Material and Methods

### Primary Human Bronchial Epithelial Cells (HBECs)

HBECs from two different study populations of adult patients with asthma (study population 1, N = 9; study population 2, N = 9), as well as from a small reference population (N = 4) of healthy subjects (**Table 1**), were obtained by bronchial brushings and expanded *in vitro*, as previously described (47). Asthma diagnosis was confirmed as defined by Global Strategy for Asthma Management and Prevention criteria, (GINA 2017; https://ginasthma.org). Asthma study population 1 was made up of steroid-free mild asthmatics, whereas study population 2 included mild to severe and uncontrolled asthmatic patients treated (N = 5) or not (N = 4) with inhaled corticosteroids (ICS). Both study populations consisted of a mix of patients with T2-high/T2-low and atopic/non-atopic phenotypes. A positive allergen specific IgE test against the 10 most common aeroallergens was used to define atopic asthma. A fractional exhaled nitric oxide (FeNO) > 25 ppb was used to define T2-high asthma. T2-low asthma is defined as FeNO < 25 ppb.

**Table 1:** Clinical and demographic characteristics of asthma patients.

	Healthy population	Study population 1	Study population 2	P-value
**N**	4	9	9	
**Demographic characteristics**				
Age [mean (range)]	22.51 (19-29)	28.56 (19-57)	42.78 (19-70)	0.217
Sex (M/F)	1/3	6/3	6/3	0.309
BMI (Kg/m^2^)	22.56 (5.01)	22.32 (3.32)	23.92 (5.06)	0.555
**Asthma phenotype**				
Atopy (Yes/No)	0/4	5/4	4/5	0.164
T2-high/T2-low	–	4/5	6/3	0.343
GINA severity*	–	9/0	4/5	0.008
**Lung function and asthma control**				
FEV1 (%)	108.5 (29.7)	96.0 (19.0)	83.0 (27.0)	0.121
FVC (%)	110.5 (37.0)	103.0 (16.5)	102.0 (26.5)	0.531
FEV1/FVC (%)	96.4 (7.0)	75.0 (13.5)	72.0 (14.5)	0.002
PD_15_ (mg)	635 (0)	178.0 (217.0)	176.4 (323.8)	0.003
ACQ_6_	–	1.50 (1.58)	1.50 (1.51)	0.616
ICS treatment (Yes/No)	–	0/9	5/4	0.008
**Blood biomarkers**				
FeNO (ppb)	–	19.0 (58.2)	25.0 (26.1)	0.730
IgE (IU/mL)	–	46.0 (162.0)	98.0 (112.5)	0.546
**Blood differential cell count**				
Leukocytes (10^3/^μL)	–	6.00 (3.20)	6.00 (2.20)	0.948
Neutrophils (10^3/^μL)	–	3.50 (1.30)	3.60 (2.30)	>0.999
Lymphocytes (10^3/^μL)	–	1.70 (0.80)	1.60 (0.85)	0.714
Monocytes (10^3^/μL)	–	0.40 (0.30)	0.60 (0.25)	0.570
Eosinophils (10^3^/μL)	–	0.10 (0.28)	0.12 (0.19)	0.812
Basophils (10^3^/μL)	–	0.04 (0.05)	0.05 (0.03)	0.502
**Sputum differential cell count**				
Sputum Eosinophils (%)	–	0.75 (8.05)	4.50 (4.50)	0.798
Sputum Neutrophils (%)	–	42.75 (64.35)	38.50 (47.25)	0.955
Sputum Macrophages (%)	–	50.63 (57.35)	46.25 (37.25)	0,955
Sputum Lymphocytes (%)	–	0.00 (0.41)	1.00 (1.00)	0,029

BMI, Body mass index; FEV1, forced expiratory volume in 1 s; FVC, forced vital capacity; PD_15_, Dose of mannitol required to reduce the FEV1 by 15% of the baseline value; ACQ_6_, Asthma Control Questionnaire; FeNO, fractional exhaled nitric oxide.

T2-high is defined as FeNO > 25 ppb.

Data are presented as median value (Interquartile range), unless otherwise expressed.

*GINA Severity: mild/moderate/severe.

Kruskal-Wallis or U-Mann Whitney test for continuous variables and chi squared test for categoric variables.

The study was conducted in accordance with the Helsinki II declaration and with permission from the Danish Committee on Health Research Ethics (Ethics: H-16043663 and H-16002008), and all patients have given informed consent.

### Design, Expression and Purification of Recombinant SARS-CoV-2 Spike Protein (SP1)

The boundaries for SP1 sequence (ID: QIA20044.1) covered aa. 325-561, surrounding the RBD. The codon-optimized gene sequence (synthesized by Geneart^©^) had a N-terminal BiP secretion signal and a C-terminal (x10) poly-histidine tag used for purification. SP1 was expressed using the ExpreS2 platform. Briefly, Schneider-2 insect cells were transfected (ExpreS2 Insect TRx5, ExpreS2ion Biotechnologies) and cells were grown at 25°C in shake flasks for 3 days. The supernatant was then centrifuged (5000 rpm; 10 min; 4°C) and filtered through a 0.22 µm vacuum filter (PES). Recombinant SP1 was further purified on a 5 mL HisTrap HP column (GE healthcare), and bound protein was eluted with 500 mM Imidazole in PBS buffer, pH 7.4.

### Imiquimod, Poly(I:C) and SP1 Treatment

HBECs (3x10^4^ cells/mL) were cultured in bronchial epithelial growth medium (BEGM, Clonetics, San Diego, CA, USA) until 80% confluence in type-I bovine collagen-coated (Advanced BioMatrix, San Diego, USA) 12-wells plates (Nunc Technolgies, Carlsbad, CA, USA) at 37°C, 5% CO_2_ in air. Cells were treated with 10 µg/mL imiquimod (Tocris Bioscience, Bristol, UK) with or without 10 µg/mL polyinosine-polycytidylic acid (poly(I:C)) (InvivoGen, San Diego, US) or SARS-CoV-2 SP1 for 3, 24 or 48 hours. Cell-free supernatants were collected for protein release analysis and cell lysates were collected for protein or gene expression analysis.

### Western Blot Analysis

Quantification of protein expression from cell lysates was performed by Western blot, as previously described (46). For that, primary rabbit anti-human antibodies from Bio-Rad, Stockholm, Sweden (ACE2 #AHP888) and Cell Signaling, Danvers, Massachusets, USA (GAPDH #5174S) were used at dilution 1:1000, and an anti-rabbit secondary antibody #09/2029 lot. 28 (Cell Signaling, Danvers, Massachusets, USA) conjugated to HRP was used. Optical density was detected using a LI-COR odyssey Fc imager system (LI-COR, Lincoln, USA). Optical density ratio between samples and GAPDH were calculated and normalized towards poly(I:C) stimulated samples.

### Sample Preparation for Mass Spectrometry

Cells were lysed in ice cold 5% SDS in 100 mM Tris (pH=7.55) sonication using a Branson Digital Sonifier^®^ 250-D (Branson Ultrasonics Corporation, Danbury, USA), at amplitude 10%, with 10s pulse on and 20s off, for a total of 36 cycles. The lysates were centrifuged at 15,871 xg for 8 minutes to remove insoluble material. 60 μg protein per cell lysate supernatant per sample was processed on HILIC Microspheres (ReSyn Biosciences, Gauteng, South Africa) in a King-Fisher Flex (Thermo Fisher Scientific, Bremen). The following steps were performed in 96-well plates in the system: magnetic microspheres (1:10 protein:beads ratio) were incubated in equilibration buffer (15% ACN, 100 mM NH_4_Ac, pH=4.5); protein samples were incubated in binding buffer (30% ACN, 200 mM NH_4_Ac, pH=4.5) for binding of proteins to HILIC beads; beads were washed twice in 95% ACN; digestion of proteins for 1h at 37°C with trypsin (20:1 protein:trypsin ratio) dissolved in 50 mM AMBIC. Peptides were recovered from the plate and dried in a Speedvac (Thermo Fisher Scientific, Germany) prior to C_18_ desalting. Peptide desalting was performed using BioPureSPN Mini, PROTO 300 C_18_ (The Nest Group, Inc., MA, USA). Briefly, columns were equilibrated with 100 μl 70% ACN, 5% FA and conditioned using 100 μl 5% FA. Peptide samples were resuspended in 100 ul 5% FA and loaded on the column. Columns were washed with 5% FA and before elution of peptides using 100 μl 50% ACN, 5% FA. The resulting peptide solution was dried by vacuum centrifugation and stored at -20°C until analysis.

### Mass Spectrometry

Samples were resuspended in 10 μl 0.1% FA and 6 μl were loaded onto an EASY-nano LC system (Thermo Fisher Scientific, Germany). The analytical column was a silica capillary (75 μm* 16 cm Pico Tip Emitter, New Objective, USA) packed in house with C18 ReproSil-Pur 1.9 μm (Dr. Maisch GmbH, Germany). Peptides were separated using a 60 min LC gradient from 5% to 25% solvent B (80% ACN, 0.1% FA) and continuously sampled by a Q-Exactive HF-X Mass Spectrometer (Thermo Fisher Scientific, Germany) through an electrospray interface. Data were acquired using data-dependent acquisition (DDA) in positive ion mode. Precursor spectra (375 to 1500 m/z) were acquired at 120,000 resolution with automatic gain control (AGC, MS1 target 3x10^6^) and a maximum injection time of 50 ms. The 20 most abundant ion peptides were continuously selected for fragmentation. Fragmentation spectra were acquired at 15,000 resolution with an AGC target of 1x10^5^ ions and a maximum injection time of 20 ms. Isolation width for fragmentation was set to 1.2 m/z.

### Knockdown of RIG-I and MDA5

For siRNA-mediated down-regulation of MDA5 and RIG-I expression, HBECs were transfected with 10 nM siRNA targeting MDA5, RIG-I or non-specific siRNA (scramble) (Ambion, Thermo Scientific, Waltham, NA, USA) using Lipofectamine RNAiMAX (*Invitrogen*, Thermo Scientific, Waltham, NA, USA), as previously described (48).

### RNA Isolation and Gene Expression Analysis Using RT-qPCR and NanoString

Total RNA was isolated from cell lysates using RNeasy Plus mini kit (Qiagen, Hilden, Germany) according to manufacturer’s protocol and 1 µg RNA was reversely transcribed into cDNA with Precision Nanoscript Reverse Transcription kit (PrimerDesign, Southampton, UK). RT-qPCR gene expression analysis was performed on an Mx3005P qPCR system (Stratagen, La Jolla, USA) with standard cycling parameters and primers from Qiagen (Stockholm, Sweden) and PrimerDesign (Chandler’s Ford, UK). GAPDH was used as reference. A full list of the primers used in qPCR analyses is shown in **Supplementary Table 1**. Gene expression was analyzed following the ΔΔCt method.

For multiplex mRNA expression analysis, the NanoString nCounter system (NanoString technologies, Seattle, USA) was used. First, RNA quality was measured using a bioanalyzer (Agilent technologies, Santa Clara, USA). Then a panel of 579 mRNAs involved in the human immune response (NanoString Immunology Human V2 panel) was analyzed. Data was normalized to seven house-keeping genes, selected using the geNorm algorithm, and differential gene expression and pathway analysis were performed. All analysis was performed with nSolver software (NanoString technologies, Santa Clara, USA) and PANTHER classification system.

### Multiplex ELISA Analysis of Released Proteins

Protein levels of IFN-β, IL-1β, TNF-α, IL-8, IL-6, IL-33, CCL-5, IL-4, IL-5, IL-13 and IL-17A were measured using a Luminex immunoassay (R&D Systems, Abingdon, UK) in cell-free supernatants on a MAGPIX instrument (R&D Systems, Abingon, UK) according to manufacturer’s instructions. Lower limit of detection was 3.86 pg/mL for IFN-β, 3.39 pg/mL for IL-1β, 11.77 pg/mL for TNF-α, 2.10 pg/mL for IL-8, 9.19 pg/mL for IL-6, 15.25 pg/mL for IL-33, and 10.12 pg/mL for IL-17A.

### Statistical Analysis

The data are presented as median (interquartile range). The normality of the data was assessed with Shapiro-Wilk test. Paired analysis of difference between groups was made using the Wilcoxon test for two group comparisons, and RM-one way ANOVA with Holm-Sidák´s *post hoc* test or Friedman with Dunn´s multiple comparisons test for more than two groups. Unpaired analyses were performed using Mann-Whitney U-test for two groups comparisons, or Kruskal-Wallis tests when multiple groups were assessed. P-values of < 0.05 were considered statistically significant. All statistical analyses were performed using GraphPad Prism version 8.0 software (GraphPad Software). Multiple t-test analyses were carried out for the detection of differentially expressed genes and proteins after Nanostring and proteomic analyses. Gene Ontology and pathway analyses were performed using the PANTHER classification system (version 13.1) and the Search Tool for the Retrieval of Interacting Genes (STRING) (Version: 11.0, www.string-db.org).

## Results

### Imiquimod Decreases ACE2 Receptor Expression and Increases IFN-β Expression in HBECs From Patients With Asthma

**Figure 1:**
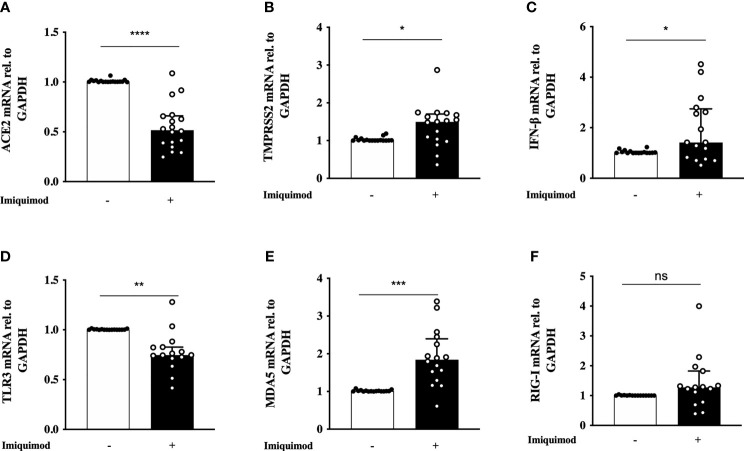
Effect of imiquimod treatment on SARS-CoV-2 receptor expression and IFN response in human bronchial epithelial cells (HBECs) from asthmatics. Imiquimod effect on the mRNA expression of ACE2 **(A)** and TMPRSS2 **(B)** in HBECs from asthmatic patients. **(C–F)** Imiquimod effect on the anti-viral response of bronchial epithelium. IFN-β mRNA expression **(C)**, as well as the pattern recognition receptors TLR3 **(D)**, MDA5 **(E)**, and RIG-I **(F)** are shown. HBECs from study population 1 and 2 were used, N = 16-18. Wilcoxon Signed Rank Test. ns, not significant; *p < 0.05; **p < 0.01; ***p < 0.001; ****p < 0.0001.

The bronchial epithelial expression of SARS-CoV-2 receptors, as well as the IFN response to virus are two key aspects of anti-viral resistance in the light of SARS-CoV-2 infection. Hence, we studied the direct effect of imiquimod on the expression of ACE2, TMPRSS2, and IFN-β signaling mediators. Treatment with imiquimod for 24 hours resulted in a clear decrease of ACE2 expression (P<0.0001) in HBECs from asthmatics compared to non-treated cells (**Figure 1A**). A slight increase in TMPRSS2 expression (P=0.0174) was, however, observed after imiquimod treatment in asthmatic epithelial cells (**Figure 1B**). Since imiquimod is known to increase IFN-β in cell systems beyond the lung (49, 50), we determined whether such an effect occurred also in HBECs. Imiquimod increased IFN-β expression (P=0.0214) in HBECs from asthmatic patients compared with untreated cells (**Figure 1C**). We also studied the effects of imiquimod on PRRs involved in dsRNA recognition (TLR3, MDA5 and RIG-I). Compared with untreated cells, imiquimod decreased TLR3 expression (P=0.0043) (**Figure 1D**). However, in accordance with the increased IFN-β expression MDA5 expression was clearly up-regulated (90% increase; P=0.0002) with imiquimod (**Figure 1E**), and no change was found in RIG-I expression (**Figure 1F**).

### Imiquimod Displays Mixed Effects on Pro-Inflammatory Response of HBECs From Steroid-Free Mild Asthmatics

**Figure 2:**
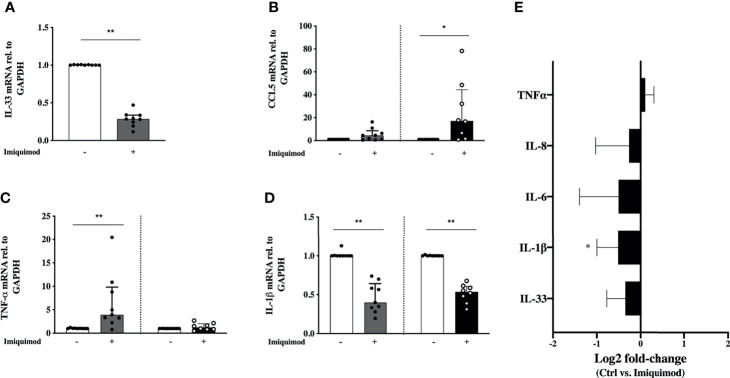
Effects of imiquimod treatment on pro-inflammatory response of human bronchial epithelial cells (HBECs) from steroid-free asthmatic patients. Imiquimod effect on the mRNA expression of the pro-inflammatory mediators IL-33 **(A)**, CCL-5 **(B)**, TNF-α **(C)** and IL-1β **(D)** is depicted. **(E)** Log2 fold-change expression of TNF-α, IL-8, IL-6, IL-1β, and IL-33 in cell culture supernatants from HBECs after 24 hours of imiquimod treatment measured by multiplex ELISA (Luminex). *p < 0.05; **p < 0.01, Wilcoxon Signed Rank Test. HBECs from study population 1 were used, N = 7-9.

Another aspect of importance in the bronchial epithelial response to virus is the viral infection tolerance. Due to potential cofounding epithelial effects of anti-inflammatory steroid treatment in asthma, the effect of imiquimod was first studied in HBECs from steroid-free mild asthmatics (Study population 1; **Table 1**). Particularly important in asthma pathophysiology, imiquimod treatment of HBECs from steroid-free asthmatic patients reduced (P<0.01) the gene expression of the allergic disease-related alarmin IL-33 (**Figure 2A**). By some contrast, the eosinophilic chemoattractant CCL5 was augmented at gene level after 3h and 24h (P<0.05) treatment with imiquimod (**Figure 2B**). However, no change was found at CCL5 protein level (data not shown). Similarly, imiquimod increased (P<0.05) TNF-α only at gene level and only after 3h imiquimod treatment (**Figure 2C**). Interestingly, imiquimod decreased (P<0.01) the gene and protein levels of IL-1β (**Figures 2D, E**), and a similar trend was found for IL-6 and IL-8 at protein level (**Figure 2E**).

### Imiquimod Treatment Modifies the Proteome of HBECs From Steroid-Free Asthmatics

**Figure 3:**
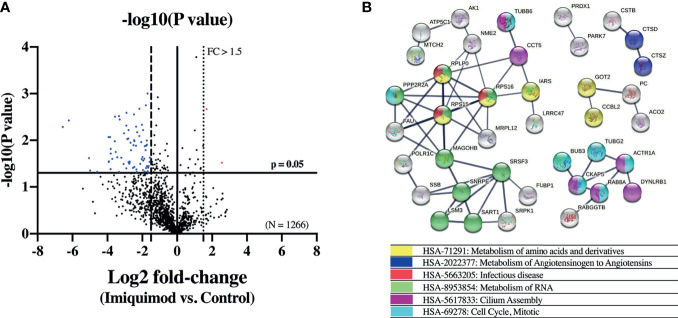
Imiquimod treatment modifies the proteome of human bronchial epithelial cells (HBECs) from patients with asthma. **(A)** Volcano plot showing differentially expressed proteins between imiquimod treated and control unstimulated HBECs from patients with steroid-free asthma (N = 3). Proteins down-regulated (blue dots; log2 fold-change < -1.5, p < 0.05) and up-regulated (red dots; log2 fold-change > 1.5, p < 0.05) are depicted. **(B)** STRING interaction network of the proteins down-regulated by imiquimod treatment. Only proteins interacting with high confidence score (0.700) are shown. Colors represent the most relevant enriched Reactome pathways.

To further explore the effect of imiquimod treatment on the bronchial epithelium immune response, a quantitative proteomic analysis was performed comparing imiquimod treated *vs* untreated HBECs from 3 steroid-free asthmatics. In this exploratory analysis, 75 out of 1266 proteins were found down-modulated with imiquimod treatment (log2 fold-change > 1.5; P < 0.05) (**Figure 3A** and **Supplementary Table 2**). Pathway analyses of these proteins indicated an enrichment of the protein pathways “*HSA-5663205: infectious diseases*” and “*HSA-168254: influenza infection*” (**Figure 3B** and **Supplementary Table 3**), which included proteins implicated in viral mRNA and protein synthesis (e.g., ribosomal proteins RPS16, RPLP0, or RPS15), highlighting a potential protective effect of imiquimod against viral infections. In line with the decreased ACE2 expression in response to imiquimod treatment, the pathway “*HSA-2022377: Metabolism of Angiotensinogen to Angiotensins”*, was also found overrepresented between the proteins most down-regulated by imiquimod (**Figure 3B** and **Supplementary Table 3**). Finally, several pathways related to metabolism of RNA, metabolism of amino acids, and cell cycle were also found enriched within down-modulated proteins in response to imiquimod treatment (**Figure 3B** and **Supplementary Table 3**). On the other hand, only 2 proteins, BLMH and PRRC2A were found up-regulated in HBECs from asthmatic patients in response to imiquimod treatment (**Supplementary Table 2**).

### Imiquimod Boosts the Interferon Response in the Presence of SP1

Furthering potential relevance of the present results with regard to COVID-19, HBECs from a study population of asthmatic patients with different severity degrees were exposed to SP1 in this study (study population 2; **Table 1**). In addition, a small reference population of healthy subjects (N = 4) was included.

The cellular entry of SARS-CoV-2 depends on the binding of SP1 to ACE2 and the subsequent priming by the protease TMPRSS2. In order to mimic, in part, the earliest phase of SARS-CoV-2 infection, we stimulated HBECs from asthma patients with SP1. We did not find any direct effect of SP1 stimulation alone (at concentrations ranging from 5nM to 100 nM) on the gene levels of ACE2, TMPRSS2, TNF-α or IFN-β in HBECs (**Supplementary Figure 1**).

**Figure 4:**
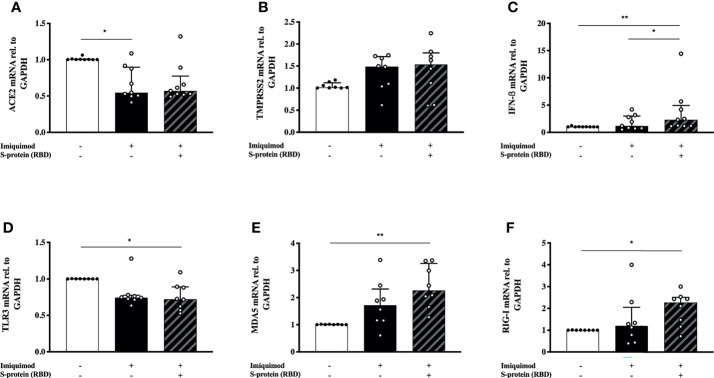
S-protein 1 (SP1) from SARS-CoV-2 in combiantion with the anti-viral drug imiquimod decreases the expression of ACE2 receptor and increases the anti-viral response in human bronchial epithelial cells (HBECs) from asthma patients. mRNA expression of ACE2 **(A)**, TMPRSS2 **(B)**, IFN-β **(C)**, and the pattern recognition receptors TLR3 **(D)**, MDA5 **(E)**, and RIG-I **(F)** in HBECs from patients with asthma (study population 2; N = 8-9) in response to imiquimod treatment alone (black bars) or in combination with SP1 (striped bars). *p < 0.05, **p < 0.01, Friedman test followed by Dunn´s multiple comparison test.

In order to address the effect of SP1 stimulation in the imiquimod treatment response of HBECs, we used a combination of SP1 with imiquimod for 24 h. There was no additional effect with imiquimod on ACE2 or TMPRSS2 gene expression in the presence of SP1 (**Figures 4A, B**). In addition, a similar trend to a decreased expression of ACE2 after imiquimod treatment (and no additional effect of SP1) was found after dividing the patients in treatment categories (i.e., ICS treated *vs*. ICS untreated) (**Supplementary Figure 2A**). However, there was a significantly higher (P<0.05) IFN-β gene expression in cells that were treated with both imiquimod and SP1 compared with cells that were only treated with imiquimod (**Figure 4C**), especially in patients treated with ICS (**Supplementary Figure 2B**). Adding SP1 did not alter the effects of imiquimod on TLR3 expression (**Figure 4D**). There was, however, a small trend to an increase of both MDA5 and RIG-I in cells treated with SP1 in combination with imiquimod compared with cells that were only treated with imiquimod (**Figures 4E, F**). Moreover, the effect of imiquimod on these parameters may not be dependent on underlying disease, as similar trends were shown for different asthma phenotypes (Atopic *vs*. Non-atopic and T2-high *vs*. T2-low; **Supplementary Figures 2C–F**) and healthy subjects (**Supplementary Figures 3A–F**).

### Imiquimod Attenuates Poly(I:C)-Induced ACE2 Upregulation and Pro-Inflammatory Response, and Boosts Poly(I:C)-Induced IFN Signaling in HBECs From Asthma Patients

**Figure 5:**
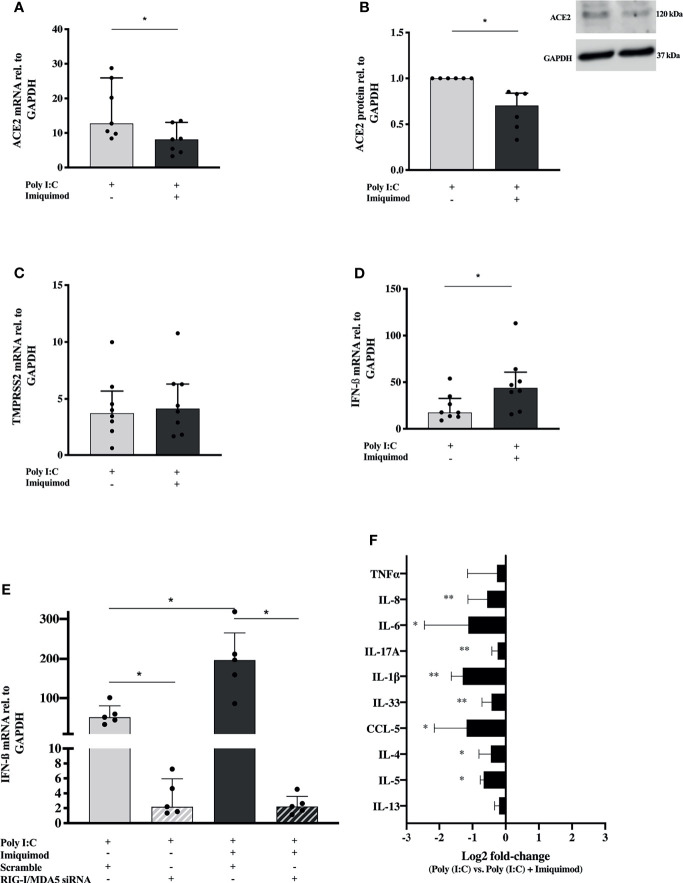
Imiquimod decreases the expression of ACE2 receptor, and several pro-inflammatory cytokines in response to poly(I:C) stimulation, but increases the IFN response in human bronchial epithelial cells (HBECs) from asthmatics. ACE2 expression in HBECs from patients with asthma after treatment with poly(I:C) alone or in combination with imiquimod at gene **(A)** and protein (N = 6) **(B)** level. TMPRSS2 **(C)**, and IFN-β **(D)** mRNA expression in HBECs from asthmatic subjects in response to poly(I:C) or in combination with imiquimod. **(E)** Imiquimod effect on poly(I:C)-induced IFN-β mRNA expression in HBECs after siRNA-mediated downmodulation of MDA5 and RIG-I expression (N = 5; RM one-way ANOVA followed by Holm-Šídák’s *post hoc* test). **(F)** Log2 fold-change [poly(I:C) *vs*. poly(I:C) + imiquimod] expression of several pro-inflammatory mediators in cell culture supernatants from HBECs after 24 hours poly(I:C) and imiquimod treatment, measured by multiplex ELISA (Luminex). Asthma (study population 2), N = 7-9 unless otherwise is expressed. Wilcoxon matched-pairs signed rank test for comparisons between two different treatment conditions. *p < 0.05; **p < 0.01.

To further characterize the effects of imiquimod on primary HBECs from study population 2 (**Table 1**) and from a small reference population of healthy subjects (N = 4), we studied its interaction with poly(I:C), a SARS-CoV-2 replication mimic that would represent biological effects of infection without being subject to variation in infection rates (18). Poly(I:C) stimulation resulted in a 10-20-fold upregulation of ACE2 mRNA expression in HBECs from asthmatic donors (**Figure 5A**). More interesting, imiquimod significantly decreased (P<0.05) poly(I:C)-induced ACE2 mRNA expression in asthmatic HBECs (**Figure 5A**), and a similar decrease was found at protein level (**Figure 5B**). Similar effects of imiquimod on ACE2 expression were shown in HBECs from healthy subjects (**Supplementary Figure 4A**). In addition, a 5-fold increase of TMPRSS2 expression was found in patients with asthma after poly(I:C) stimulation, but no effect of imiquimod was found on TMPRSS2 expression in unstimulated asthmatic or healthy HBECs (**Figure 5C** and **Supplementary Figure 4B**).

Imiquimod further increased (P<0.05) the poly(I:C)-induced IFN-β gene expression in asthmatic HBECs (**Figure 5D**). Following siRNA mediated down-modulation of MDA5 and RIG-I mRNAs (**Supplementary Figures 5A, B**), poly(I:C)-induced IFN-β was inhibited and the imiquimod-dependent effect on IFN-β expression was absent (**Figure 5E**), suggesting a role of these PRRs in poly(I:C) and imiquimod mediated IFN upregulation.

The imiquimod-dependent down-regulation of ACE2 expression did not differ between ICS treated and untreated patients, or between different asthma phenotypes (**Supplementary Figures 6A–C**). No changes in the imiquimod-dependent upregulation of IFN-β expression were found between ICS treated and untreated patients, or between different asthma phenotypes (**Supplementary Figures 6D–F**).

Expanding gene expression data, we wanted to investigate the effect of imiquimod on the release of poly(I:C)-induced pro-inflammatory cytokines involved in COVID-19 pathogenesis. Importantly, in the presence of the viral infection mimic, imiquimod significantly decreased the majority of the cytokines evaluated and none were increased (**Figure 5F**).

### Imiquimod Treatment of Poly(I:C)-Stimulated HBECs Resulted in Upregulation of Multiple Anti-Viral Gene Expression Pathways

**Figure 6:**
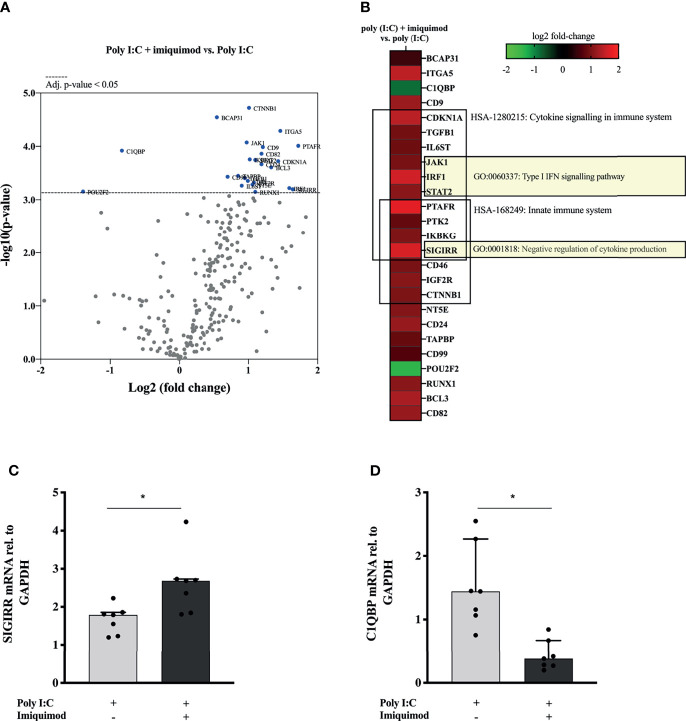
Imiquimod induces the upregulation of multiple anti-viral gene expression pathways in human bronchial epithelial cells (HBECs) exposed to the viral replication mimic poly(I:C). **(A)** Volcano plot showing differentially expressed genes between poly(I:C) + imiquimod treated *versus* poly(I:C)-stimulated HBECs, N = 3. Multiple t-test analyses with the corrected method of Benjamini and Yekutieli were used. An adjusted p-value < 0.05 was considered significant. **(B)** Heat map representation of the 25 most down-regulated and up-regulated genes in response to imiquimod treatment. The most relevant overrepresented Gene Ontology (GO) terms are depicted. N = 3. SIGIRR **(C)** and C1QBP **(D)** mRNA expression in HBECs from asthmatic subject (N = 7) in response to poly(I:C) or in combination with imiquimod. Wilcoxon matched-pairs signed rank test for comparisons between two different treatment conditions. *p < 0.05.

Finally, we explored the impact of imiquimod treatment on the HBECs response to poly(I:C) by performing a multiplex mRNA expression analysis of a total number of 579 genes involved in the human immune response. 68 of these genes were found differentially expressed in response to imiquimod treatment with an adjusted P-value < 0.1 (**Figure 6A** and **Supplementary Table 4**). Reactome pathways overrepresentation of the 25 most up-regulated and down-regulated genes (P < 0.05) against human whole genome is shown in **Table 2**. In line with our previous findings, differential expression analyses revealed a major effect of imiquimod treatment in several genes from innate immune pathways (**Figure 6B** and **Table 2**). Importantly, several genes involved in type-I IFN signaling pathway (GO:0060337), including the IRF1, JAK1, and STAT2, were up-regulated (P < 0.05) in poly(I:C)-stimulated HBECs after imiquimod treatment (**Figure 6B**). Other IFN-stimulated genes, such as IFN alpha/beta receptor (IFNAR1, IFNAR2), positive regulators of IFN-beta production (e.g., IRF5), and other anti-viral genes (e.g., STAT1, STAT2) also tend to increase (adjusted P < 0.1) after imiquimod treatment (**Supplementary Table 4**). In the same line, C1QBP, encoding a negative regulator of DDX58- and IFIH1-mediated signaling pathways (gC1qR), was found down-regulated with imiquimod treatment in HBECs stimulated with poly (I:C) (**Figure 6B**). On the other hand, SIGIRR, encoding a negative regulator of IL-1R signaling, was shown to be up-regulated by imiquimod treatment (**Figure 6B**), potentially explaining the imiquimod-mediated down-modulation of IL-1ß and IL-33 shown in **Figures 2**, **5**. Finally, both SIGIRR expression and C1QBP expression effects of imiquimod were further validated by using RTqPCR in HBECs from study population 2 (**Figures 6C, D** and **Table 1**).

**Table 2:** Reactome pathways overrepresentation against whole human genome.

	Observed	Expected	Fold Enrichment	FDR
**Immune System (R-HSA-168256)**	16	2.62	6.11	2.89E-02
**Cytokine Signaling in Immune system (R-HSA-1280215)**	10	1.00	10.01	4.07E-07
**Hemostasis (R-HSA-109582)**	7	.81	8.62	4.00E-02
**Innate Immune System (R-HSA-168249)**	7	1.34	5.22	5.17E-03
**Transcriptional Regulation by RUNX3 (R-HSA-8878159)**	4	.11	35.43	4.43E-02
**Interferon Signaling (R-HSA-913531)**	4	.24	16.81	2.49E-02
**Apoptosis (R-HSA-109581)**	4	.21	19.27	3.60E-02
**Interferon Gamma Signaling (R-HSA-877300)**	3	.11	27.16	1.51E-02
**Interleukin-4 and Interleukin-13 signaling (R-HSA-6785807)**	3	.13	22.26	1.26E-02
**Transcriptional regulation by RUNX2 (R-HSA-8878166)**	3	.14	21.12	4.27E-02
**Interleukin-27 Signaling (R-HSA-9020956)**	2	.01	>100	3.08E-03
**Interleukin-35 Signaling (R-HSA-8984722)**	2	.01	>100	2.36E-05
**IL-6-type Cytokine Receptor Ligand Interactions (R-HSA-6788467)**	2	.02	96.92	1.56E-02
**Interleukin-20 Family Signaling (R-HSA-8854691)**	2	.03	65.90	1.71E-02
**Antigen Presentation: Folding, Assembly and Peptide Loading of Class I MHC (R-HSA-983170)**	2	.03	63.37	2.72E-02

## Discussion

**Figure 7:**
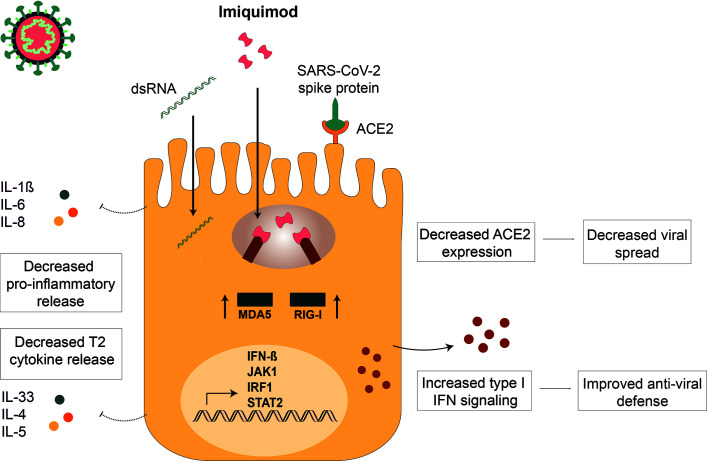
Imiquimod treatment exerts anti-viral actions in human bronchial epithelium. Imiquimod exert dual actions on human bronchial epithelium: 1) Improves viral resistance by decreasing ACE2 expression (decreased viral spread) and increasing IFN-β expression (restored viral defense) on human bronchial epithelial cells (HBECs). 2) Potentiates viral infection tolerance by reducing viral stimulus-induced epithelial cytokines involved in severe COVID-19 infection (Prevention of cytokine storm).

There is an urgent need of finding new drugs for the treatment of respiratory viral infections in asthma. In this study, several pharmacological effects of the TLR7 agonist imiquimod on HBECs from asthmatics, of interest for the treatment of both viral induced asthma exacerbations and COVID-19, have been demonstrated (**Figure 7**). On one hand, imiquimod boosts IFN-β response and decreases ACE2 expression in HBECs both at baseline and in the presence of the viral infection mimics poly(I:C) and the spike protein of the SARS-CoV-2, SP1. On this note, multiplex gene expression profiling of poly(I:C)-stimulated HBECs subjected to imiquimod treatment demonstrated change of multiple genes involved in anti-viral immune response. We further observed a decrease of C1QBP, a negative regulator of MDA5 and RIG-I signaling. Proteomic analysis of HBECs also highlighted that the potential protective effect of imiquimod on viral infections involved a down-modulation of proteins implicated in viral mRNA and protein synthesis. On the other hand, our results evidenced a down-modulation of several pro-inflammatory mediators implicated in both viral-induced asthma exacerbations and COVID-19 pathophysiology following imiquimod treatment. This was in part supported by the up-regulation of SIGIRR, a negative regulator of TLR signaling, in poly (I:C)-stimulated HBECs treated with imiquimod.

Increasing evidence demonstrate that severe asthma is a risk factor for COVID-19 related admission to intensive care and death (8, 9). Indeed, a deficient anti-viral interferon response in asthma patients has been previously described, potentially leading to more severe virus-induced exacerbations (25). Yuen et al. have also shown that SARS-CoV-2 directly suppresses the interferon response during infection *in vitro* (51). Treatment with nebulized IFN-β in COVID-19 patients led to greater odds of improvement compared with patients receiving placebo control (52). The same treatment in asthma patients has been shown to reduce need of additional treatment while boosting innate immunity (53). However, IFN-β therapy was mainly beneficial in patients with severe asthma, whereas side effects occurred in milder asthmatics (53). It is possible that drugs boosting endogenous production of IFN-β may hold the advantage of potentially avoiding flu-like symptoms normally associated with exogenous interferon treatment (54). The present data extend previous observations on imiquimod-induced type I IFN in diverse experimental models to primary HBECs (37, 55, 56). Of particular interest, our data support the feasibility of obtaining drugs that improve airway IFN-β levels both at baseline and during a viral infection. mRNA expression profiling of imiquimod´s effect on poly(I:C)-stimulated HBECs revealed upregulation of multiple genes involved in type-I IFN pathway including STAT1, STAT2, IRF1, and JAK1. In addition, a decrease in C1QBP mRNA expression, a negative regulator of MDA5/RIG-I signaling (57), was found after imiquimod treatment of HBECs also exposed to poly (I:C). Further, siRNA mediated inhibition of MDA5/RIG-I expression in HBECs inhibited the imiquimod-induced effect on IFN-β expression, thus highlighting a potential role of these PRRs in imiquimod mediated IFN upregulation. A similar RIG-I and STAT1-dependent upregulation of type I IFN in response to imiquimod has been previously described in plasmacytoid dendritic cells (pDCs) (56).

Apart from the impaired IFN response in asthma and COVID-19 patients, another possible factor for developing a severe respiratory SARS-CoV-2 infection is the rapid increase of ACE2 expression in the first hours of SARS-CoV-2 infection, probably leading to an exaggerated replication and a rapid spread (58). Supporting the relevance of the present work, we demonstrated that poly(I:C) also increased ACE2 in bronchial epithelial cells from asthma and healthy donors. Given the importance of ACE2 levels in initiation and development of the SARS-CoV-2 infection, it is of interest that pharmacological attenuation of ACE2 expression levels may decrease the infection rates of the virus (58). Finney et al. have shown a glucocorticoid-induced lowering of the levels of ACE2 in the bronchial epithelium, strongly arguing that this action is caused by steroid-induced suppression of type I interferon (28). Previous studies have further shown that ACE2 could be an interferon-stimulated gene in airway epithelium (59). By contrast, our study demonstrates that imiquimod both reduces poly(I:C)-induced ACE2 expression and increases IFN-β in HBECs. These distinct observations are not readily explained by different sources of HBECs, COPD (28) and asthma (this study). Further work is clearly warranted to explain the dual effects on infection resistance of imiquimod, increasing IFN-β and decreasing ACE2, which would co-operate in reducing SARS-CoV-2 infectivity.

Both SARS-CoV-2 infection and viral-induced asthma exacerbations display hyperinflammatory immune responses, indicating a dysfunctional host tolerance to infection (60). In this study, imiquimod decreased gene expression of cytokines of special interest in asthma pathogenesis such as IL-33 and IL-1β. Furthering potential relevance of imiquimod treatment in asthma, Maazi et al. have previously demonstrated that TLR7 activation on pDCs induces the release of IFN-α, which in turn will inhibit the proliferation and cytokine production in ILC2 cells, key mediators of asthma pathogenesis (61). Imiquimod was also suggested to reduce allergen-induced airway inflammation in mice, potentially triggering T1 responses and decreasing T2 inflammation (62). Moreover, IL-33 protein release was also reduced by imiquimod in HBECs after poly (I:C) stimulation in the present study. Stanczak et al., have recently demonstrated that IL-33 expression is increased in COVID-19 seropositive patients after stimulation with SARS-CoV-2 peptides (63). They also evidenced that disease severity and T-cell activation in SARS-CoV-2 infected subjects is correlated with IL-33 production (63). Indeed, IL-33 has been purposed as a key player in the pathogenesis of COVID-19, possibly being involved in early activation of innate immune responses, but also in later stages of the disease by inducing lung fibrosis (64). Other cytokines and chemokines such as IL-1β, IL-6, IL-8, or TNF-α have also been implicated in the pathogenesis of viral-induced exacerbations of asthma and severe COVID-19 (17, 65). We demonstrated that IL-1β, IL-6 and IL-8 protein expression were reduced by imiquimod in HBECs at poly(I:C) stimulation. Imiquimod transiently increased baseline gene expression of TNF-α, but this action was not reflected by protein release; in presence of poly(I:C), TNF-α protein was rather reduced by imiquimod. Taken together, the present data suggest that imiquimod-like drugs targeting SARS-CoV-2 infected airway epithelium are of interest for further development towards tentative treatment of viral induced exacerbation of asthma and COVID-19 both at early stages to avert infection and at later stages associated with airway hyperinflammation. It is not known to what extent the present profile of action of imiquimod is shared with other TLR7 agonists, including two drugs that already have been subjected to clinical trials to determine anti-allergic efficacy by local airway administration in asthma and rhinitis (41, 42).

Imiquimod-induced attenuation of the pro-inflammatory cytokines IL-6 and TNF-α, the neutrophilic chemokine IL-8, or different TH-type cytokines including IL-4, IFN-β, or IL-17, has been previously shown in airway epithelial cells or mouse models of RSV and Influenza A virus infections (37, 38). Of note, Salinas et al. suggested the interference of imiquimod with viral macromolecular synthesis as one potential mechanism of imiquimod-dependent down-regulation of pro-inflammatory cytokines after RSV infection (38). This possibility is in line with our exploratory proteomic analysis on the direct effect of imiquimod on HBECs; our data revealed a possible protective effect of imiquimod on viral infections through down-modulation of proteins implicated in viral mRNA and protein synthesis, including several ribosomal proteins. In addition, multiplex mRNA expression analyses in this study demonstrated an increased expression of the negative regulator of IL-1R signaling SIGIRR in imiquimod treated HBECs, highlighting another potential mechanism of the imiquimod-mediated down-modulation of IL-1β and IL-33 expression (66).

Imiquimod is currently available as a drug, Aldara, for topic treatment of skin conditions, including genital warts, actinic keratosis, and superficial basal cell carcinoma. Delivery of imiquimod has previously been employed in mice against influenza A infection (37), and for reducing airway inflammation in rats (62). Moreover, efficacy, as well as safety profile of intranasal application of imiquimod have been also demonstrated in rhinovirus infection in primates (67). However, this study has demonstrated an unexpected profile of action of imiquimod indicating drug feasibility rather than suggesting that imiquimod would be an ideal drug for nasal or inhalational treatment in COVID-19 or future viral pandemics.

A limitation in this study is the use of poly(I:C) and SP1 as a mimic of SARS-CoV-2 and rhinovirus infection, but not the real virus. Poly(I:C) is a dsRNA analogue and TLR3 agonist that mimics several biological effects of viral infection without being subject to the variation in intensity that may apply to actual infections. Poly(I:C) stimulation of bronchial epithelial cells induces a similar cytokine profile compared to rhinovirus and SARS-CoV-2 infection in bronchial epithelial cells (18, 68, 69). Moreover, poly(I:C) and SP1 administration to mice has been proven to induce cytokine storm syndrome and ARDS (70). Although studies on interaction between imiquimod and actual infection by SARS-CoV-2 are now warranted we argue that our approach employing poly(I:C) has strong guiding merits. In this study we have used submerged and not differentiated human bronchial epithelial cells. Ravindra et al. have previously demonstrated that basal bronchial epithelial cells are infected over the course of SARS-CoV-2 infection (71). We further demonstrate that our undifferentiated bronchial basal cell-like monolayer harbors significant ACE2 receptors and respond well to poly(I:C). Moreover, infectivity by rhinovirus is also particularly pronounced in human bronchial basal cells (72). Furthermore, undifferentiated epithelium commonly occurs in asthmatic bronchi, which is a focus in our study. It reflects both preferential shedding of columnar differentiated cells and actual denudation where the naked basement membrane is promptly covered by basal cell-like repair cells.

In conclusion, we demonstrate that imiquimod increases IFN-β expression, in part targeting MDA5 and RIG-I receptors and involving activation of several anti-viral immune pathways. In addition, we demonstrate, for the first time, that treatment with imiquimod reduced ACE2 expression in the bronchial epithelium. Finally, the drug´s profile would be additionally beneficial through its anti-inflammatory effect, reducing several epithelium-derived cytokines and chemokines implicated in the hyper-inflammatory response in viral-induced asthma exacerbation and severe COVID-19 cases. Taken together, the present demonstration of several epithelial actions of imiquimod in HBECs of potential importance for human respiratory viral infections is both promising and challenging. The promise lies in demonstration of feasibility of finding drugs with multiple desirable actions in virus-induced asthma exacerbations and COVID-19. The challenge is manifold but in part lies in further delineation of mechanisms of action of a drug with the present profile of action.

## Data Availability Statement

The datasets presented in this study can be found in online repositories. The names of the repository/repositories and accession number(s) can be found below: Proteomic data have been uploaded to the PRIDE database (73) under accession no: PXD029135; Nanostring data has been uploaded to NCBI GEO under accession no: GSE181281.

## Ethics Statement

The studies involving human participants were reviewed and approved by Danish Committee on Health Research Ethics (Ethics: H-16043663 and H-16002008). The patients/participants provided their written informed consent to participate in this study.

## Author Contributions

JJN-F contributed to the design, acquisition, analysis, and interpretation of data, have drafted and revised the work. ST contributed to the design, acquisition, analysis, and interpretation of data, have drafted and revised the work. SC contributed to the design, acquisition, analysis, and interpretation of data, have drafted and revised the work. AS contributed to the acquisition, analysis, and interpretation of data, and have revised the work. MH contributed to the acquisition, analysis, and interpretation of data, and have revised the work. SR contributed to the acquisition, analysis, and interpretation of data, and have revised the work. MM contributed to acquisition, analysis, and interpretation of data, and have revised the work. AFS contributed to the design of the work, interpretation of data, and revised the work. CP contributed to the design, analysis, and interpretation of data, have drafted and revised the work. LU contributed to the design, acquisition, analysis, and interpretation of data, have drafted and revised the work. All authors contributed to the article and approved the submitted version.

## Funding

This research has been funded by Vetenskapsrådet (Swedish medical research council) grant numbers: 2020-00922_VR and 2017-00806_VR as well as Swedish Heart and lung foundation grant number: 20180207_HLF.

## Conflict of Interest

MH reports personal fees from Astra Zeneca, personal fees from Novartis, outside the submitted work. CP reports grants and personal fees from AZ, grants and personal fees from Novartis, grants and personal fees from GlaxoSmithKline, grants and personal fees from TEVA, grants and personal fees from GSK, grants and personal fees from Chiesi, grants from Pharmaxis, grants and personal fees from Sanofi, outside the submitted work.

The remaining authors declare that the research was conducted in the absence of any commercial or financial relationships that could be construed as a potential conflict of interest.

## Publisher’s Note

All claims expressed in this article are solely those of the authors and do not necessarily represent those of their affiliated organizations, or those of the publisher, the editors and the reviewers. Any product that may be evaluated in this article, or claim that may be made by its manufacturer, is not guaranteed or endorsed by the publisher.

## Abbreviations

ACE2, Angiotensin Converting Enzyme II; BEGM, Bronchial Epithelial Growth Medium; COVID-19, Coronavirus Disease-19; dsRNA, Double Stranded RNA; HBECs, Human Primary Bronchial Epithelial Cells; ICS, Inhaled Corticosteroids; IRFs, Interferon Regulatory Factors; MDA-5, Melanoma Differentiation-Associated protein 5; PRRs, Pattern Recognition Receptors; RBD, Receptor Binding Domain; RIG-I, Retinoic Acid-Inducible Gene I; SARS-CoV-2, Severe Acute Respiratory Syndrome-Related Coronavirus 2; SP1, Spike Protein 1; STRING, Search Tool for the Retrieval of Interacting Genes; ssRNA, Single Stranded RNA; TLR, Toll-Like Receptor; WHO, World Health Organization.
