# Supplementary material for: Imiquimod Boosts Interferon Response, and Decreases ACE2 and Pro-Inflammatory Response of Human Bronchial Epithelium in Asthma
Source: Front Immunol. 2021 Dec 7;12:743890. doi: 10.3389/fimmu.2021.743890 (PMC8688760; doi:10.3389/fimmu.2021.743890)
Supplement: Supplementary file 1 [file DataSheet_1.pdf]

TITLE: Imiquimod increases interferon response, and decreases ACE2 and pro-inflammatory response of human bronchial epithelium in asthma.

Juan José Nieto-Fontarigo, PhD[a†], Sofia Tillgren, MSc[a†], Samuel Cerps, MSc[a], Asger Sverrild, MD, PhD[b], Morten Hvidtfeldt, MD, PhD[b], Sangeetha Ramu, MSc[a], Mandy Menzel, PhD[a], Adam Frederik Sander, PhD[c,d], Celeste Porsbjerg, MD, PhD[b] and Lena Uller, PhD[a*]

a. Department of Experimental Medical Science, BMC D12, Lund University, Sweden

b. Department of Respiratory Medicine, University Hospital Bispebjerg, Copenhagen, Denmark

c. Department for Immunology and Microbiology, Faculty of Health and Medical Sciences, Centre for Medical Parasitology, University of Copenhagen, Denmark

d. Department of Infectious Disease, Copenhagen University Hospital, Denmark

†These authors have contributed equally to this work and share first authorship

SUPPLEMENTARY TABLES

Supplementary Table 1. List of primers used in qPCR assays.

Primer	Provider	Cat. No or sequence
**ACE2**	Qiagen	#PPH02572A-200
**CCL-5**	Primer Design	Sense: -AACCCAGCAGTCGTCTTTGTC-<br>Anti-sense: -AGCAAGCAGAAACAGGCAAAT-
**GAPDH**	Qiagen	#PPH00150F-200
**IFN-ß**	Qiagen	#PPH00384F-200
**IL-1ß**	Qiagen	#PPH00171C-200
**IL-33**	Primer Design	Sense: -AAAGAAAGATAAGGTGTTACTGAGTTA-<br>Anti-sense: -GCAACCAGAAGTCTTTTGTAGG-
**MDA5**	Qiagen	#PPH18927A-200
**RIG-I**	Qiagen	#PPH20774A-200
**TLR3**	Qiagen	#PPH01803E-200
**TMPRSS2**	Qiagen	#PPH02262C-200
**TNF-α**	Primer Design	Sense: -AGGTTCTCTTCCTCTCACATAC-<br>Anti-sense: -ATCATGCTTTCAGTGCTCATG-

Supplementary Table 2. Differentially expressed proteins in imiquimod treated HBECs compared with unstimulated cells.					
**Proteins**	**Log2 Fold-Change**	**SE of difference**	**P value**	**t ratio**	**df**
**SNRPE**	-6,56171	0,89168	0,005187	7,35881	3
**SRSF3**	-6,21941	0,382767	0,003766	16,2486	2
**HIST1H1D**	-5,04973	1,19302	0,02413	4,23273	3
**LSM3**	-4,99512	1,49896	0,04464	3,33239	3
**LRRC47**	-4,58984	1,03937	0,047644	4,416	2
**KPNA2**	-3,9309	0,815816	0,008533	4,81837	4
**UNC45A**	-3,92184	0,995889	0,029174	3,93803	3
**DDB1**	-3,91515	0,672336	0,028247	5,82321	2
**MYO1E**	-3,85071	0,889308	0,049416	4,33001	2
**RPLP0**	-3,84009	0,622533	0,008576	6,16849	3
**FAU**	-3,82132	1,11485	0,041609	3,42765	3
**PDLIM5**	-3,81022	0,760234	0,015292	5,0119	3
**PPP2R2A**	-3,80562	0,826075	0,019235	4,60687	3
**RPS16**	-3,7158	1,0621	0,024929	3,49854	4
**SERPINH1**	-3,58003	0,62466	0,010546	5,73117	3
**IARS**	-3,56413	0,406114	0,003116	8,77618	3
**GLOD4**	-3,52306	0,744378	0,017879	4,73289	3
**POLR1C**	-3,43976	0,53216	0,023108	6,46378	2
**PC**	-3,33423	0,184187	0,003038	18,1024	2
**H2AFY**	-3,32857	1,17777	0,04753	2,82615	4
**KYAT3**	-3,29688	0,394455	0,003591	8,35806	3
**NME2**	-3,20386	0,901682	0,02373	3,55321	4
**PPM1G**	-3,08191	0,813783	0,032286	3,78714	3
**OLA1**	-3,04894	0,6011	0,007119	5,07226	4
**ALDH2**	-2,92441	0,387685	0,00483	7,54328	3
**SART1**	-2,91746	0,837691	0,039976	3,48274	3
**RPS15**	-2,91108	0,310919	0,00258	9,36281	3
**SSRP1**	-2,86601	0,48037	0,009421	5,96625	3
**PARP9**	-2,80669	0,270853	0,009185	10,3624	2
**HADHA**	-2,7977	0,844861	0,029615	3,31144	4
**EPHA2**	-2,79283	0,40058	0,006055	6,97197	3
**PRDX4**	-2,74692	0,500466	0,031627	5,48873	2
**MRPL12**	-2,74321	0,690695	0,028534	3,97167	3
**DYNLRB1**	-2,72754	0,693046	0,029222	3,93558	3
**FGFBP1**	-2,68276	0,603694	0,0113	4,44391	4
**RABGGTB**	-2,652	0,342202	0,016246	7,74981	2

**EHBP1L1**	-2,55026	0,725696	0,039079	3,51423	3
**ACTR1A**	-2,50933	0,468417	0,012727	5,35704	3
**CSTB**	-2,48053	0,377541	0,002777	6,57021	4
**PARK7**	-2,474	0,53713	0,009986	4,60596	4
**BAG6**	-2,45961	0,48795	0,037176	5,04069	2
**PYGB**	-2,45741	0,540879	0,01047	4,54337	4
**AK1**	-2,37158	0,274356	0,01312	8,64419	2
**ACO2**	-2,36188	0,319264	0,005108	7,3979	3
**DNM1L**	-2,34205	0,795705	0,042247	2,94337	4
**SRPK1**	-2,32568	0,526564	0,04763	4,41672	2
**ARPC1B**	-2,29883	0,342638	0,006757	6,70923	3
**GOT2**	-2,1734	0,307206	0,002107	7,07473	4
**FUBP1**	-2,1709	0,444056	0,008109	4,88881	4
**MTCH2**	-2,11918	0,388677	0,032031	5,45229	2
**PPAT**	-2,06297	0,217776	0,010961	9,4729	2
**CKAP5**	-2,03639	0,58553	0,025401	3,47786	4
**TUBB6**	-2,01976	0,264456	0,004661	7,63741	3
**PCYT1A**	-1,99888	0,555935	0,022849	3,59553	4
**ATP5F1C**	-1,96519	0,58294	0,043373	3,37117	3
**PARP4**	-1,95957	0,152864	0,00603	12,8191	2
**SSB**	-1,95447	0,57453	0,027231	3,40186	4
**CTSZ**	-1,88564	0,450758	0,024894	4,18327	3
**DNAJB1**	-1,8711	0,659607	0,047025	2,83669	4
**LRPPRC**	-1,85979	0,439302	0,013332	4,23352	4
**ANXA5**	-1,81793	0,456096	0,016321	3,98585	4
**CHMP4A**	-1,80368	0,244101	0,001789	7,38909	4
**STK26**	-1,79284	0,51221	0,039475	3,5002	3
**AKR1D1**	-1,7871	0,207206	0,013178	8,62474	2
**IRGQ**	-1,76979	0,554029	0,049544	3,19441	3
**BUB3**	-1,74554	0,177051	0,010132	9,85898	2
**TCOF1**	-1,74135	0,402874	0,012425	4,32232	4
**PRDX1**	-1,7398	0,559818	0,035949	3,10779	4
**CTSD**	-1,70007	0,595924	0,046264	2,85282	4
**YME1L1**	-1,69816	0,0536669	0,000997	31,6427	2
**CCT5**	-1,68679	0,601648	0,048632	2,80361	4
**TUBG2**	-1,66527	0,471709	0,038631	3,5303	3
**RAB8A**	-1,6408	0,450583	0,035704	3,64151	3
**TES**	-1,58368	0,400785	0,028917	3,95144	3
**MAGOHB**	-1,56455	0,244117	0,023491	6,409	2
**PCBP1**	-1,49743	0,330137	0,010531	4,53578	4
**COL17A1**	-1,48815	0,465558	0,033011	3,19649	4
**EIF4G1**	-1,44462	0,432599	0,028852	3,33939	4

**CCT2**	-1,42648	0,443983	0,032499	3,21291	4
**RPL7**	-1,40494	0,441398	0,033441	3,18293	4
**STRN4**	-1,38507	0,00536675	0,000015	258,083	2
**CHMP2A**	-1,38029	0,458552	0,039546	3,01012	4
**TSTA3**	-1,3568	0,29575	0,019453	4,58765	3
**RPSA**	-1,35606	0,481385	0,047974	2,817	4
**UGDH**	-1,3109	0,265262	0,007806	4,94188	4
**NCL**	-1,29884	0,465118	0,049187	2,79249	4
**H1FX**	-1,28915	0,34366	0,033088	3,75125	3
**ZPR1**	-1,28088	0,145179	0,012604	8,82276	2
**RPL15**	-1,27365	0,371481	0,041582	3,42857	3
**AARS**	-1,27151	0,434016	0,042829	2,92964	4
**RPL35**	-1,22423	0,356583	0,026457	3,43323	4
**PMPCB**	-1,20445	0,0837988	0,004806	14,3731	2
**ZC3H18**	-1,19632	0,342199	0,039596	3,49597	3
**CCT3**	-1,18026	0,363484	0,031463	3,24706	4
**S100A14**	-1,17765	0,379394	0,03608	3,10403	4
**LUC7L2**	-1,1344	0,32618	0,040118	3,47782	3
**TAGLN2**	-1,11943	0,136366	0,0012	8,20901	4
**MTHFD1**	-1,09515	0,31604	0,025695	3,46523	4
**NCEH1**	-1,06112	0,0488548	0,002113	21,72	2
**CAPRIN1**	-0,995311	0,140708	0,019406	7,07358	2
**HNRNPK**	-0,979516	0,289114	0,027581	3,38799	4
**PTMA**	-0,97186	0,335491	0,044259	2,89682	4
**ANXA1**	-0,961044	0,344685	0,049404	2,78818	4
**EHD1**	-0,861052	0,242568	0,038098	3,54973	3
**PCBP2**	-0,839867	0,300174	0,048914	2,79794	4
**RPS4X**	-0,7765	0,259901	0,040431	2,98768	4
**HSPB1**	-0,755001	0,240647	0,034936	3,13738	4
**EZR**	-0,719886	0,23859	0,039269	3,01725	4
**NOLC1**	-0,692483	0,173517	0,016253	3,99087	4
**APEX1**	-0,673531	0,227833	0,041709	2,95625	4
**NUP93**	-0,59549	0,136017	0,011893	4,37806	4
**GOLGA3**	-0,566146	0,113512	0,037928	4,98754	2
**KPNA1**	-0,540556	0,0812648	0,021862	6,65178	2
**SFN**	-0,52892	0,182967	0,044528	2,89079	4
**PAPOLA**	0,190326	0,0360134	0,033989	5,28487	2
**ITGA3**	0,301182	0,0592206	0,01469	5,08577	3
**ASPH**	0,628595	0,207584	0,038851	3,02815	4
**MTHFS**	0,719578	0,11672	0,025316	6,16498	2
**KIF2A**	0,773129	0,277441	0,049482	2,78664	4
**LSM12**	0,889807	0,204151	0,048817	4,35858	2

**ESYT2**	0,997048	0,14355	0,006121	6,94567	3
**MAPK3**	1,03331	0,27319	0,032391	3,78238	3
**LPCAT1**	1,08621	0,0139707	0,000165	77,7491	2
**GNG12**	1,10427	0,276352	0,016185	3,99588	4
**FAM120A**	1,19085	0,287768	0,025616	4,13823	3
**OXSR1**	1,33251	0,448985	0,041233	2,96782	4
**CUL4B**	1,34495	0,382956	0,039141	3,51202	3
**SDC4**	1,48367	0,28102	0,013251	5,27959	3
**BLMH**	1,67269	0,0776476	0,002148	21,5421	2
**PRRC2A**	2,55132	0,455423	0,030418	5,60208	2

Supplementary Table 3. Reactome pathways overrepresented in imiquimod downmodulated proteins.						
**#term ID**	**term description**	**observed gene count**	**background gene count**	**strength**	**FDR**	**matching proteins in your network**
**HSA-1430728**	Metabolism	19	2032	0.39	0.0296	ACO2,PYGB,AKR1D1,GOT2,RPS16,CCBL2,LRPPRC,ALDH2,PPAT,PCYT1A,ATP5C1,PARP9,AK1,IARS,HADHA,PARP4,PC,RPLP0,RPS15
**HSA-2408522**	Selenoamino acid metabolism	4	112	0.97	0.0296	RPS16,IARS,RPLP0,RPS15
**HSA-71291**	Metabolism of amino acids and derivatives	6	354	0.65	0.0471	GOT2,RPS16,CCBL2,IARS,RPLP0,RPS15
**HSA-2022377**	Metabolism of Angiotensinogen to Angiotensins	2	17	1.49	0.0471	CTSZ,CTSD
**HSA-1852241**	Organelle biogenesis and maintenance	7	287	0.8	0.0296	CCT5,RAB8A,TUBB6,ATP5C1,DYNLRB1,ACTR1A,CKAP5
**HSA-380320**	Recruitment of NuMA to mitotic centrosomes	4	91	1.06	0.0296	TUBG2,TUBB6,ACTR1A,CKAP5
**HSA-68877**	Mitotic Prometaphase	5	190	0.84	0.0296	TUBG2,TUBB6,BUB3,ACTR1A,CKAP5
**HSA-69275**	G2/M Transition	6	189	0.92	0.0296	TUBG2,RAB8A,TUBB6,PPP2R2A,ACTR1A,CKAP5
**HSA-68886**	M Phase	6	343	0.66	0.0471	TUBG2,TUBB6,PPP2R2A,BUB3,ACTR1A,CKAP5
**HSA-69278**	Cell Cycle, Mitotic	7	483	0.58	0.0471	TUBG2,RAB8A,TUBB6,PPP2R2A,BUB3,ACTR1A,CKAP5
**HSA-75153**	Apoptotic execution phase	3	51	1.19	0.0296	HIST1H1D,MST4,DNM1L
**HSA-3371497**	HSP90 chaperone cycle for steroid hormone receptors (SHR)	3	53	1.17	0.0297	DNAJB1,TUBB6,ACTR1A
**HSA-168254**	Influenza Infection	4	148	0.85	0.0471	RPS16,KPNA2,RPLP0,RPS15
**HSA-5663205**	Infectious disease	6	363	0.63	0.0471	RPS16,SSRP1,CHMP4A,KPNA2,RPLP0,RPS15
**HSA-8953854**	Metabolism of RNA	9	652	0.56	0.0296	RPS16,LSM3,SART1,MAGOHB,PPP2R2A,SRSF3,SNRPE,RPLP0,RPS15

**HSA-73856**	RNA Polymerase II Transcription Termination	3	65	1.08	0.0471	MAGOHB,SRSF3,SNRPE
**HSA-975957**	Nonsense Mediated Decay (NMD) enhanced by the Exon Junction Complex (EJC)	5	112	1.07	0.0296	RPS16,MAGOHB,PPP2R2A,RPLP0,RPS15
**HSA-109688**	Cleavage of Growing Transcript in the Termination Region	3	65	1.08	0.0471	MAGOHB,SRSF3,SNRPE
**HSA-72163**	mRNA Splicing - Major Pathway	5	178	0.86	0.0296	LSM3,SART1,MAGOHB,SRSF3,SNRPE
**HSA-197264**	Nicotinamide salvaging	2	19	1.44	0.0471	PARP9,PARP4
**HSA-5617833**	Cilium Assembly	6	195	0.9	0.0296	CCT5,RAB8A,TUBB6,DYNLRB1,ACTR1A,CKAP5

Supplementary Table 4. Differentially expressed genes (p-value < 0.1) in poly(I:C) stimulated cells treated with imiquimod vs. poly(I:C) stimulated cells.

mRNA	Log2 fold change	std error (log2)	Lower confidence limit (log2)	Upper confidence limit (log2)	P-value	BY.p.value	probe.ID
**CTNNB1**	1.01	0.083	0.846	1.17	1.89e-05	0.0225	NM_001098210.1:1815
**BCAP31**	0.547	0.0484	0.453	0.642	2.84e-05	0.0225	NM_005745.7:495
**ITGA5**	1.46	0.143	1.18	1.74	5.13e-05	0.0262	NM_002205.2:925
**JAK1**	0.976	0.104	0.771	1.18	8.47e-05	0.0262	NM_002227.1:285
**PTAFR**	1.72	0.188	1.35	2.09	9.75e-05	0.0262	NM_000952.3:1035
**CD9**	1.21	0.134	0.949	1.47	0.000103	0.0262	NM_001769.2:405
**C1QBP**	-0.831	0.0945	-1.02	-0.645	0.00012	0.0262	NM_001212.3:745
**CD82**	1.19	0.139	0.919	1.46	0.000137	0.0262	NM_002231.3:1211
**IKBKG**	1.02	0.124	0.773	1.26	0.000175	0.0262	NM_003639.2:470
**STAT2**	1.1	0.134	0.836	1.36	0.000179	0.0262	NM_005419.2:1965
**CDKN1A**	1.43	0.176	1.08	1.77	0.000189	0.0262	NM_000389.2:1975
**PTK2**	0.833	0.104	0.63	1.04	2E-04	0.0262	NM_005607.3:1005
**CD24**	1.19	0.151	0.897	1.49	0.000216	0.0262	NM_013230.2:95
**BCL3**	1.33	0.172	0.993	1.67	0.000247	0.0279	NM_005178.2:450
**TAPBP**	0.852	0.118	0.621	1.08	0.000356	0.0367	NM_003190.4:1536
**CD99**	0.701	0.0978	0.51	0.893	0.000371	0.0367	NM_002414.3:625
**TGFB1**	0.948	0.134	0.686	1.21	0.000394	0.0367	NM_000660.3:1260
**CD46**	0.992	0.143	0.712	1.27	0.000443	0.0389	NM_172350.1:365
**IGF2R**	1.08	0.158	0.772	1.39	0.00048	0.0399	NM_000876.1:2605
**NT5E**	1.06	0.157	0.752	1.37	0.000521	0.0411	NM_002526.2:1214
**IL6ST**	0.904	0.135	0.639	1.17	0.000545	0.0411	NM_002184.2:2505
**IRF1**	1.59	0.242	1.11	2.06	0.000605	0.0435	NM_002198.1:510
**SIGIRR**	1.64	0.254	1.15	2.14	0.000638	0.0439	NM_021805.2:469
**POU2F2**	-1.39	0.218	-1.82	-0.962	0.000702	0.0449	NM_002698.2:908
**RUNX1**	1.1	0.172	0.757	1.43	0.00071	0.0449	NM_001754.4:635
**BCL10**	0.723	0.118	0.493	0.954	0.00085	0.0517	NM_003921.2:1250
**STAT6**	0.35	0.0578	0.236	0.463	0.000925	0.0529	NM_003153.3:2030
**ARG2**	1.07	0.177	0.72	1.41	0.000936	0.0529	NM_001172.3:1150
**PLAU**	1.09	0.184	0.725	1.45	0.00105	0.0573	NM_002658.2:793
**IL13RA1**	0.853	0.146	0.567	1.14	0.00111	0.0574	NM_001560.2:1230
**TGFBR1**	1.45	0.249	0.961	1.94	0.00113	0.0574	NM_004612.2:4280
**MYD88**	0.93	0.162	0.613	1.25	0.00121	0.0589	NM_002468.3:2145

**NOTCH2**	0.87	0.152	0.571	1.17	0.00125	0.0589	NM_024408.3:2842
**MAPK1**	0.566	0.0994	0.371	0.761	0.00127	0.0589	NM_138957.2:430
**ICAM1**	2.02	0.362	1.31	2.73	0.00142	0.0641	NM_000201.2:2253
**ITGB1**	0.749	0.136	0.482	1.02	0.00151	0.0657	NM_033666.2:2000
**MCL1**	0.669	0.122	0.43	0.909	0.00154	0.0657	NM_021960.3:1260
**IRF5**	2.51	0.463	1.6	3.41	0.00165	0.0685	NM_002200.3:1845
**IFNAR1**	0.979	0.182	0.622	1.33	0.00169	0.0685	NM_000629.2:3123
**PTGS2**	-1.13	0.212	-1.55	-0.717	0.00175	0.069	NM_000963.1:495
**CD3EAP**	-2.03	0.381	-2.77	-1.28	0.00179	0.069	NM_012099.1:555
**C14orf166**	0.112	0.0212	0.0707	0.154	0.00185	0.0695	NM_016039.2:210
**CD44**	0.713	0.135	0.447	0.978	0.00189	0.0696	NM_001001392.1:429
**PRKCD**	0.839	0.161	0.523	1.15	0.00199	0.0706	NM_006254.3:2165
**PSMB7**	0.306	0.0589	0.191	0.422	0.00202	0.0706	NM_002799.2:420
**EGR1**	1.83	0.356	1.14	2.53	0.00211	0.0706	NM_001964.2:1505
**STAT1**	0.745	0.145	0.461	1.03	0.00214	0.0706	NM_007315.2:205
**FYN**	1.23	0.238	0.758	1.69	0.00214	0.0706	NM_002037.3:765
**NFKBIA**	0.946	0.187	0.579	1.31	0.00232	0.0746	NM_020529.1:945
**RELB**	0.968	0.194	0.588	1.35	0.00247	0.0746	NM_006509.2:250
**ILF3**	-0.308	0.0618	-0.429	-0.187	0.00249	0.0746	NM_001137673.1:730
**CD164**	0.545	0.11	0.329	0.761	0.00258	0.0746	NM_006016.4:2575
**CASP3**	0.422	0.0853	0.254	0.589	0.0026	0.0746	NM_032991.2:685
**STAT3**	0.737	0.149	0.444	1.03	0.00263	0.0746	NM_139276.2:4535
**SMAD3**	0.628	0.128	0.377	0.88	0.00272	0.0746	NM_005902.3:4220
**CSF1**	1.29	0.264	0.773	1.81	0.00273	0.0746	NM_000757.4:823
**MALT1**	0.728	0.149	0.436	1.02	0.00276	0.0746	NM_006785.2:909
**STAT4**	1.17	0.239	0.7	1.64	0.00276	0.0746	NM_003151.2:789
**IFNAR2**	1.13	0.232	0.675	1.58	0.00278	0.0746	NM_000874.3:631
**TNFSF15**	1.58	0.332	0.927	2.23	0.00314	0.0829	NM_001204344.1:2338
**PECAM1**	0.866	0.184	0.505	1.23	0.00332	0.0855	NM_000442.3:1365
**CD58**	0.606	0.129	0.353	0.86	0.00338	0.0855	NM_001779.2:478
**RELA**	0.714	0.153	0.415	1.01	0.00341	0.0855	NM_021975.2:360
**LTB4R2**	-1.04	0.222	-1.47	-0.6	0.00346	0.0855	NM_019839.4:1250
**LITAF**	0.734	0.159	0.421	1.05	0.00368	0.0895	NM_004862.3:1000
**HLA-DMA**	1.03	0.225	0.587	1.47	0.00384	0.0919	NM_006120.3:380
**TRAF6**	0.659	0.146	0.373	0.945	0.00404	0.0954	NM_145803.1:1839
**CCL20**	1.79	0.399	1.000	2.57	0.0042	0.0977	NM_004591.1:35

SUPPLEMENTARY FIGURES

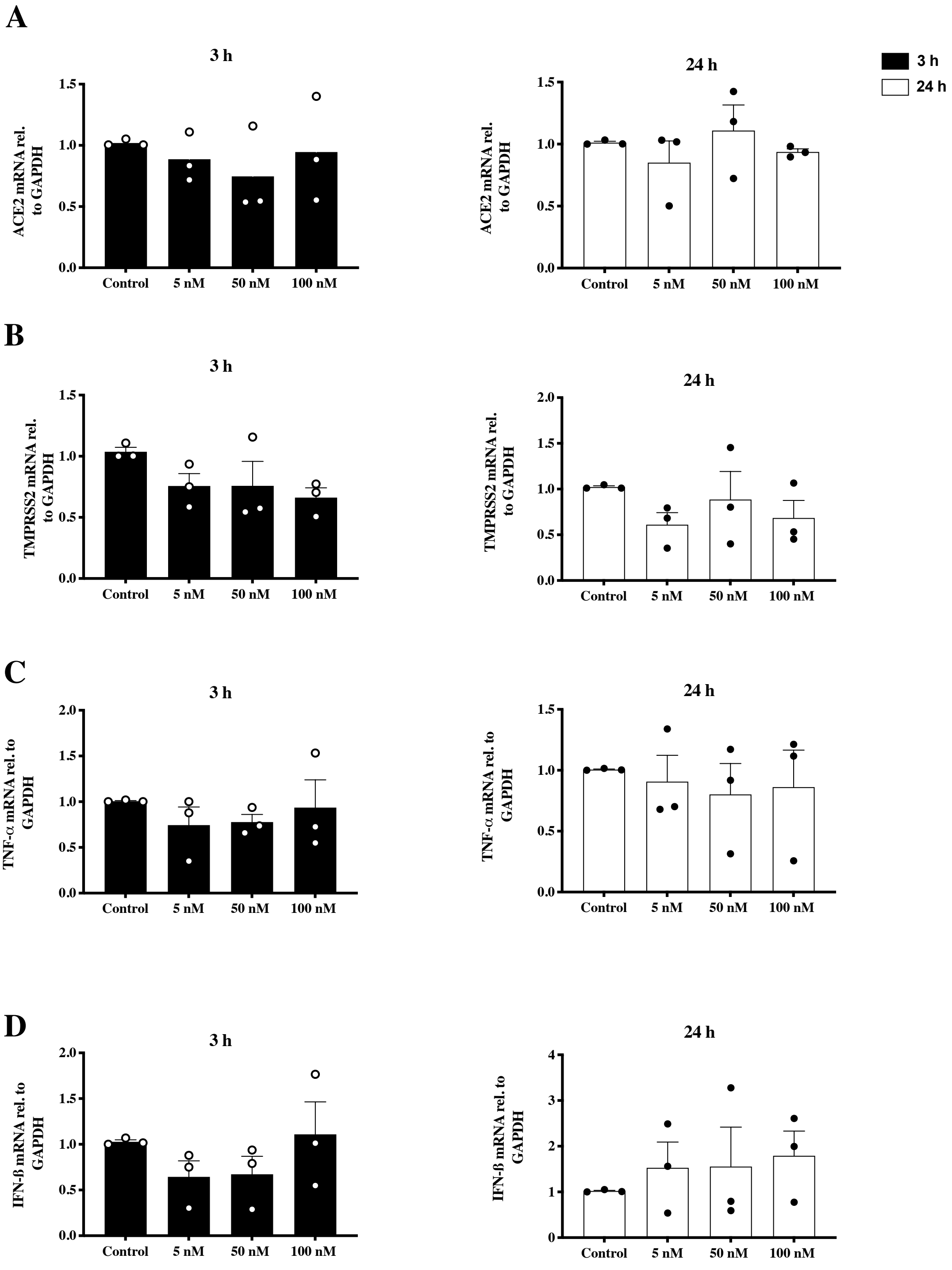

Supplementary Figure 1. SARS-CoV-2 S-protein 1 (SP1) effect on human bronchial epithelial cells from asthmatic donors. Dose response (5-100 nM) mRNA expression of the SARS-CoV-2 receptor ACE2 (A), the host protease TMPRSS2 (B), the proinflammatory cytokine TNF-α (C), and the type I interferon IFN-β (D) after 3h (left) and 24h (right) stimulation with SP1. N = 3.

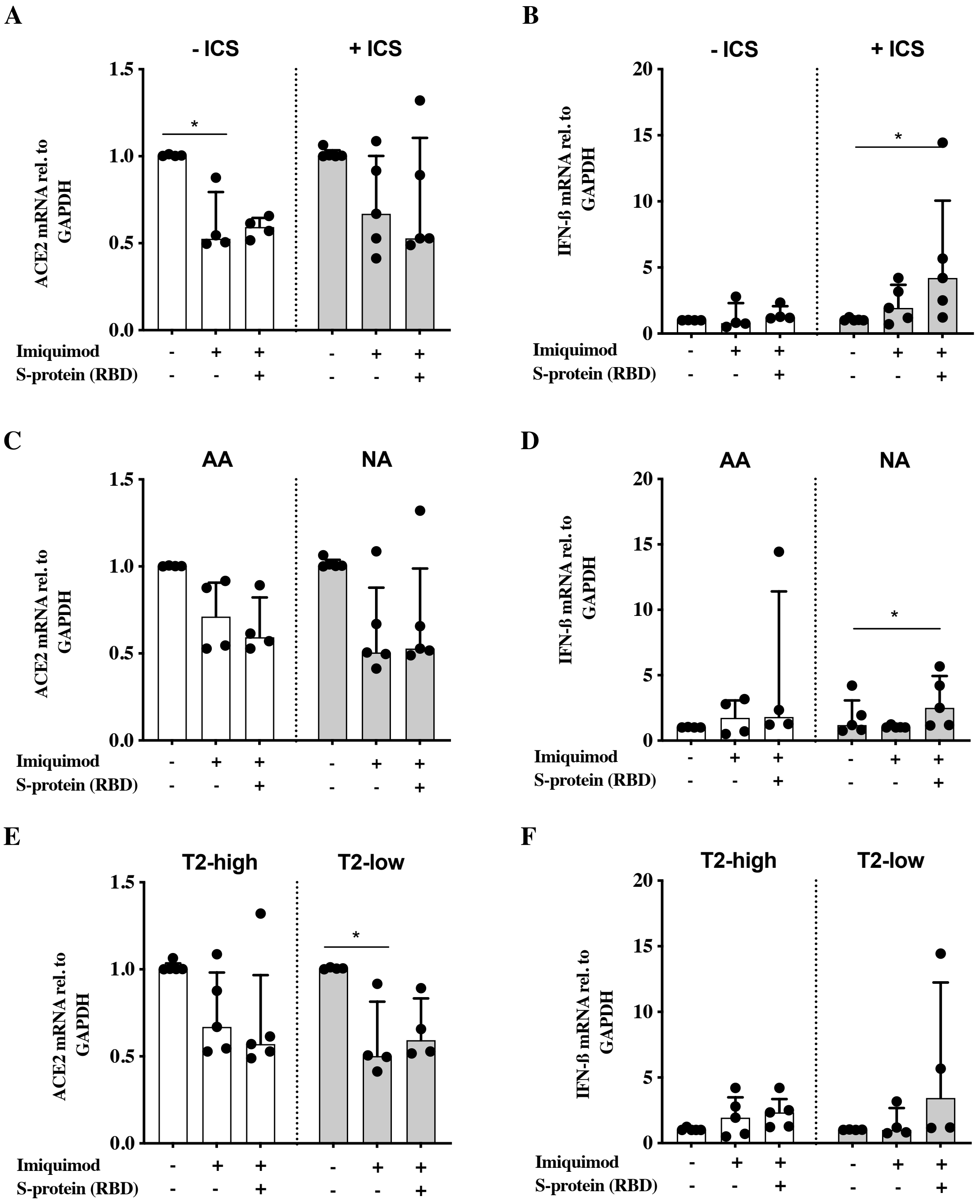

Supplementary Figure 2. Imiquimod trend to decrease the expression of ACE2 receptor and trend to increase IFN-β response independently of asthma phenotype. mRNA expression of ACE2 (A) and IFN-β (B) in HBECs from patients with asthma (cohort 2) treated (N = 5) or not (N = 4) with inhaled corticosteroids (ICS) in response to imiquimod treatment alone or in combination with SP1. mRNA expression of ACE2 (C) and IFN-β (D) in HBECs from patients with allergic (AA; N = 4) or non-allergic asthma (NA; N = 5) phenotypes in response to imiquimod treatment alone or in combination with SP1. mRNA expression of ACE2 (E) and IFN-β (F) in HBECs from patients with T2-high (N = 5) or T2-low (N = 4) phenotypes in response to imiquimod treatment alone or in combination with SP1. * $p < 0.05$, Friedman test followed by Dunn´s multiple comparison test.

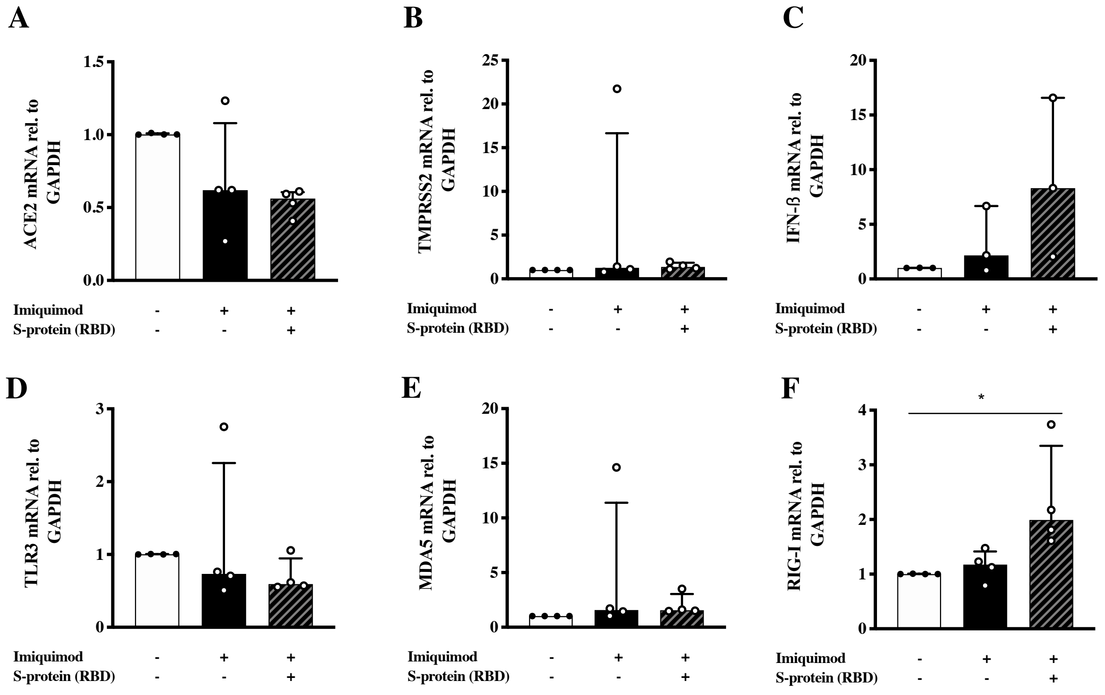

Supplementary Figure 3. S-protein 1 (SP1) from SARS-CoV-2 in combination with imiquimod trend to decrease the expression of ACE2 receptor and increase the anti-viral response in human bronchial epithelial cells (HBECs) from healthy subjects. mRNA expression of ACE2 (A), TMPRSS2 (B), IFN-β (C), and the pattern recognition receptors TLR3 (D), MDA5 (E), and RIG-I (F) in HBECs in response to imiquimod treatment alone (black bars) or in combination with SP1 (striped bars). * $p < 0.05$, Friedman test followed by Dunn´s multiple comparison test. N = 4.

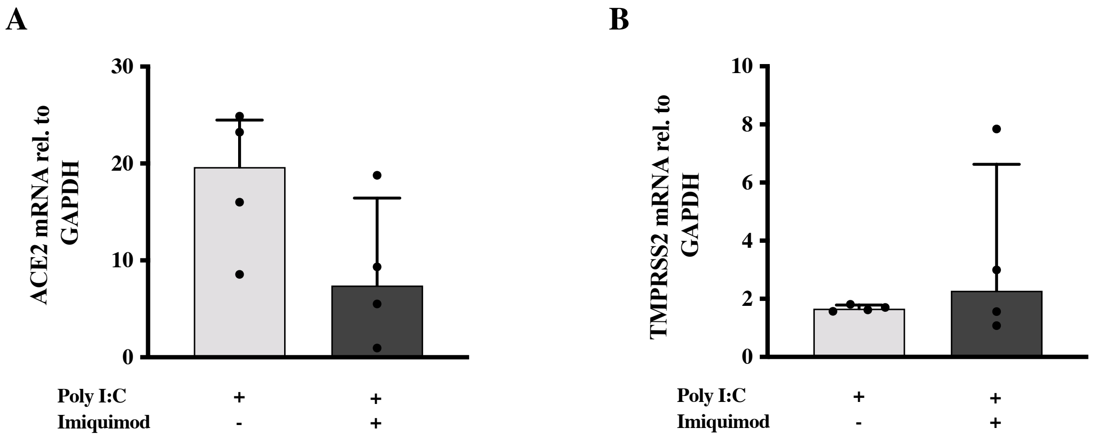

Supplementary Figure 4. Imiquimod trends to decrease the poly(I:C)-induced expression of ACE2 receptor, but not TMPRSS2 on human bronchial epithelial cells (HBECs) from healthy subjects. Effect of imiquimod treatment in the poly(I:C) induced ACE2 (A) and TMPRSS2 (B) expression in HBECs from healthy subjects (N = 4).

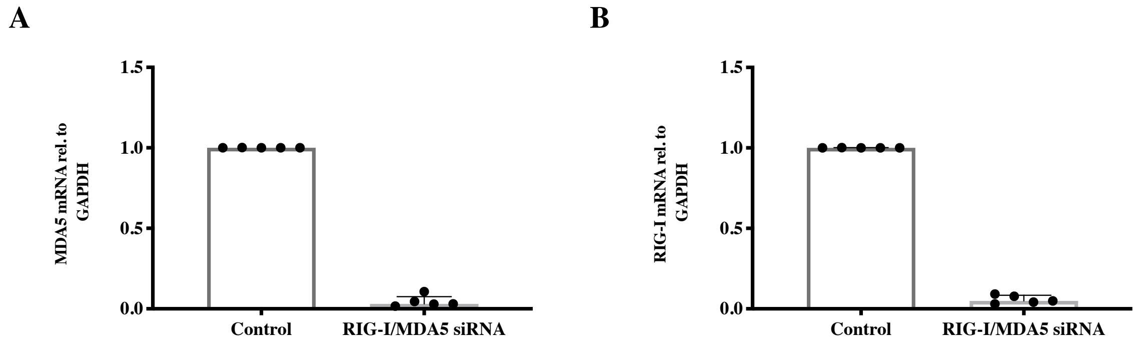

Supplementary Figure 5. siRNA knock-down of MDA5 and RIG-I mRNA. MDA5 (A) and RIG-I (B) mRNA expression in HBECs relative to control cells after siRNA-mediated downmodulation of MDA5 and RIG-I. N = 5.

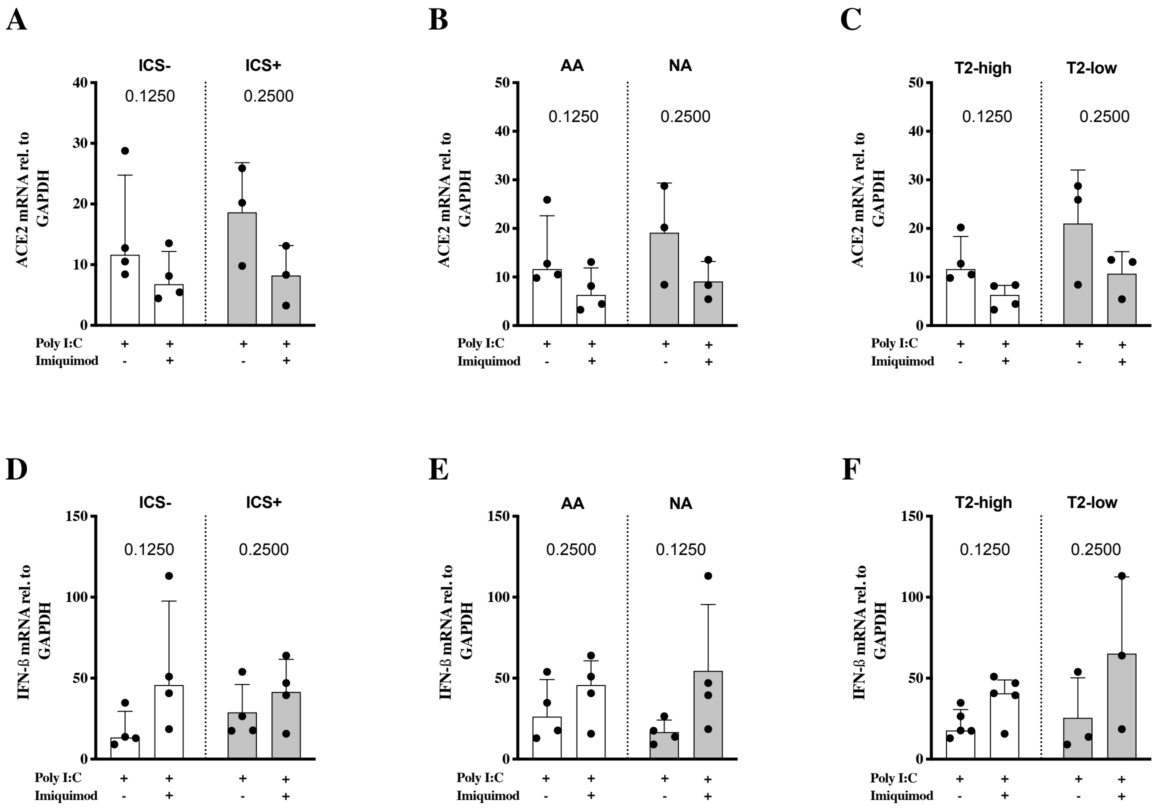

Supplementary Figure 6. Imiquimod trend to decrease the poly(I:C)-induced expression of ACE2 receptor, whereas trend to increase the poly(I:C)-induced IFN-β response independently of asthma phenotype. (A) mRNA expression of ACE2 after poly(I:C) stimulation of HBECs from patients with asthma (cohort 2) treated (N = 3) or not (N = 4) with inhaled corticosteroids (ICS) in response to imiquimod treatment. (B-C) mRNA expression of ACE2 after poly(I:C) stimulation of HBECs from patients with allergic (AA; N = 4) / non-allergic asthma (NA; N = 4) (B) or T2-high (N = 4) / T2-low (N = 3) asthma (C) in response to imiquimod treatment. (D) mRNA expression of IFN-β after poly(I:C) stimulation of HBECs from patients with asthma treated or not with ICS in response to imiquimod treatment. (E-F) mRNA expression of IFN-β after poly(I:C) stimulation of HBECs from patients with AA / NA asthma (B) or T2-high / T2-low asthma (C) in response to imiquimod treatment. Number on the graph represent p-values (Wilcoxon test).
